# ESCCAL-1 promotes cell-cycle progression by interacting with and stabilizing galectin-1 in esophageal squamous cell carcinoma

**DOI:** 10.1038/s41698-022-00255-x

**Published:** 2022-03-01

**Authors:** Yuanbo Cui, Ming Yan, Wei Wu, Pengju Lv, Jinwu Wang, Yanping Huo, Yanan Lou, Xiwen Ma, Jing Chang, Fangxia Guan, Wei Cao

**Affiliations:** 1 Translational Medicine Center, Zhengzhou Central Hospital Affiliated to Zhengzhou University, Zhengzhou, 450007 China; 2 School of Life Sciences, Zhengzhou University, Zhengzhou, 450001 China; 3 Basic Medical College, Zhengzhou University, Zhengzhou, 450001 China; 4 Department of Medicine, University of California, San Francisco, CA USA; 5 Department of Pathology, Linzhou Cancer Hospital, Linzhou, 456550 China; 6 Department of Breast Surgery, Zhengzhou Central Hospital Affiliated to Zhengzhou University, Zhengzhou, 450007 China; 7 Jiangsu Mai Jian Biotechnology Development Company, Wuxi, 214135 China

**Keywords:** Oncogenes, Cell growth

## Abstract

Long non-coding RNAs (LncRNAs) play important roles in the development of human esophageal squamous cell carcinoma (ESCC). Our previous studies have shown that knockdown of LncRNA ESCCAL-1 expression inhibits the growth of ESCC cells, but the mechanisms remain largely unknown. In this study, we show that over-expression of ESCCAL-1 promotes ESCC cell proliferation and cell-cycle progression by blocking ubiquitin-mediated degradation of an oncoprotein galectin-1 (Gal-1). Multiple LncRNA expression datasets as well as our own data together reveal that ESCCAL-1 is evidently up-regulated in ESCC tissues and exhibits promising diagnostic value. Over-expression of ESCCAL-1 augmented ESCC cell proliferation and cell-cycle progression, whereas down-regulation of ESCCAL-1 resulted in the opposite effects. Mechanistically, LncRNA ESCCAL-1 directly binds to Gal-1 and positively regulates its protein level without affecting its mRNA level. Up-regulation of Gal-1 facilitated ESCC cell proliferation and cell-cycle progress. Knockdown of Gal-1 mitigated the effects of ESCCAL-1-mediated high cellular proliferation, NF-κB signaling activation and tumorigenicity of ESCC cells. Thus, our findings provide novel insight into the mechanism by which ESCCAL-1 facilitates ESCC tumorigenesis and cell-cycle progression by interacting with and stabilizing Gal-1 protein, suggesting a potential therapeutic target for ESCC.

## Introduction

Esophageal cancer (ESCA) is one of the most frequently diagnosed malignancies globally and is the sixth leading cause of cancer-induced death worldwide^1,2^. Influenced by lifestyle, genetics and other factors, ESCC is the predominant histopathological subtype of ESCA in China, rather than esophageal adenocarcinoma (EAC)^3–5^. Despite significant advances in cancer diagnosis and treatment in recent decades, there is a lack of effective clinical treatment strategies for ESCA. In fact, according to the Global Cancer Statistics Report 2012^2^ and 2020^6^, the number of new cases of ESCA increased from 323,000 to 604,100, and deaths from 281,200 to 544,076 each year. Moreover, the five-year overall survival (OS) rate for ESCA patients is only about 20%, even in developed countries^1,7,8^. This is mainly due to tumor recurrence and uncontrolled growth driven by key molecules^9,10^. Therefore, identifying the key molecules that regulate ESCA progression and revealing the mechanisms of action may provide novel insights for the development of effective diagnostic markers and therapeutic targets.

Aberrant activation of cell proliferation signals is one of the hallmarks of tumor progression. Inhibition of cell proliferation by blocking cell-cycle process is widely considered to be an effective clinical treatment strategy for cancer^11,12^. Galectin-1 (Gal-1) protein, encoded by the *LGALS1* gene, is widely over-expressed and plays a crucial role in the development of tumors, such as lung cancer, upper urinary urothelial carcinoma, and neuroendocrine carcinomas^13–15^. Gal-1 promotes tumor progression by modulating various biological functions such as proliferation, migration, angiogenesis, and resistance to immunotherapy-induced apoptosis^16–18^. Pituitary tumor transforming gene (PTTG) induced Gal-1 trans-activation and expression promotes tumor cell motility and metastasis in ESCC^19^. But the expression pattern and specific roles of Gal-1 in ESCC remain largely unknown.

The role of non-coding RNAs, especially lncRNAs, in tumorigenesis and progression has drawn increasing attention^20,21^. LncRNAs function as oncogenes or tumor suppressors to modulate tumor cell proliferation in a tumor type-dependent manner^22,23^. Numerous studies have revealed that lncRNAs promote tumor progression via gene regulation at the transcriptional level, post-transcriptional level, and post-translational level^21^. In particular, the list of lncRNAs implicated in the initiation and development of ESCC is gradually expanding^24,25^. We have previously identified an ESCC-associated lncRNA, ESCCAL-1, that is significantly over-expressed in ESCC relative to adjacent normal tissues. Our recent multi-omics studies uncovered that DNA demethylation at the promoter of ESCCAL-1 and increase of YY-1 transcription factor binding is responsible for the upregulation of ESCCAL-1 in ESCC^26,27^. As lncRNAs mainly transcriptionally regulate target genes, but lncRNAs modulate protein ubiquitination have been reported^12,25^. Therefore, we hypothesized that interaction between ESCCAL-1 and Gal-1 protein may facilitate ESCC progression and explore the possibility of lncRNA-mediated stability of oncogenic protein Gal-1 in tumors.

In this study, we demonstrated that ESCCAL-1 directly binds to Gal-1 protein and potentiates its stability by blocking SMAD-specific E3 ubiquitin protein ligase 1 (Smurf1)-mediated ubiquitination. Moreover, ESCCAL-1 cooperates with Gal-1 to promote ESCC proliferation and cell-cycle progression, suggesting that disruption of their interaction may provide novel insight for the diagnosis and treatment of ESCC.

## Results

### Multiple transcriptional datasets reveal ESCCAL-1 overexpression as a potential diagnostic biomarker for ESCC

**Fig. 1:**
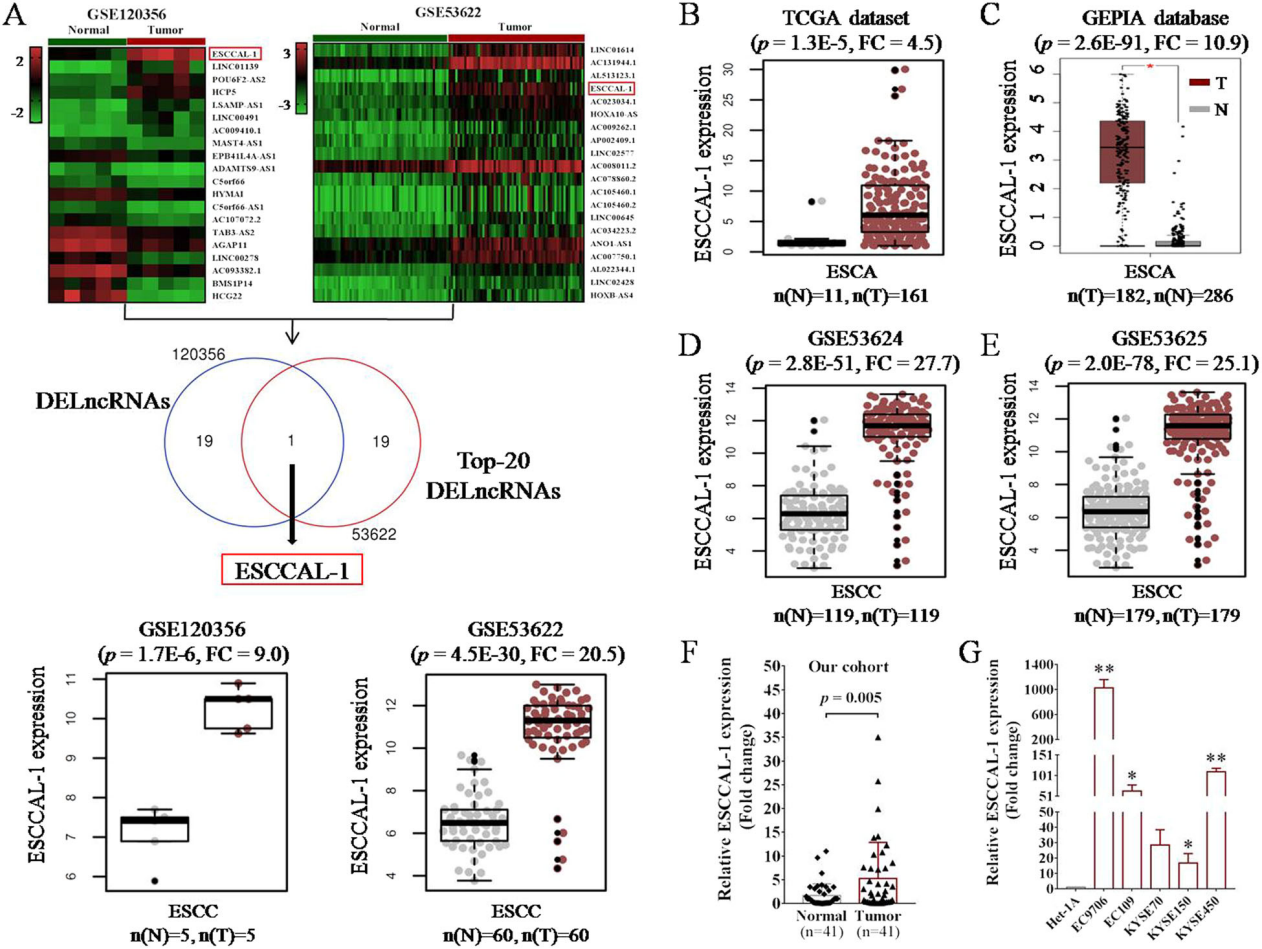
Multiple transcriptional datasets reveal the overexpression of LncRNA ESCCAL-1 in human ESCC tissues. **A** Identification of ESCCAL-1 as one of the most upregulated LncRNAs in ESCC tissues from GSE120356 and GSE53622 datasets. Heat maps showed the Top-20 differential expressed LncRNAs (DELncRNAs) between ESCC tumor tissues and the matched normal tissues from GSE120356 (5 pairs of tissue samples) and GSE53622 (60 pairs of tissue samples). The Venn diagram at the middle shows ESCCAL-1 is the only DELncRNA among the two datasets. Box plots at the bottom display ESCCAL-1 expression in normal and tumor tissues from the two datasets, respectively. **B** The expression of ESCCAL-1 in 11 cases of normal tissues and 161 cases of ESCA tissues was analyzed from the TCGA dataset. **C** The expression of ESCCAL-1 in 286 cases of normal tissues and 182 cases of ESCA tissues was analyzed from the GEPIA dataset. **D**, **E** The expression of ESCCAL-1 in paired ESCC tissue samples was analyzed from the GSE53624 (119 pairs of tissue samples) and GSE53625 (179 pairs of tissue samples) datasets. **F** The expression of ESCCAL-1 in 41 pairs of ESCC tissues was determined by qRT-PCR. Fold change (FC) is ESCCAL-1 expression in tumors over normal tissues. **G** The expression of ESCCAL-1 in 5 ESCC cell lines (EC9706, EC109, KYSE70, KYSE150, and KYSE450) and one normal epithelial cell line (Het-1A) was determined by qRT-PCR, *p < 0.05 or **p < 0.01 as compared to Het-1A.

By analyzing the LncRNA expression data of GSE120356 (5 pairs of ESCC specimens) and GSE53622 (60 pairs of ESCC specimens) from GEO, we found that ESCCAL-1 was one of the most significantly upregulated lncRNAs in ESCC compared to the matched normal tissues (Fig. 1A). Additional four independent gene expression datasets from ESCC samples were then analyzed, including TCGA cohort (11 normal and 161 tumor samples, GEPIA cohort (286 normal and 182 tumor specimens), GSE53624 (119 paired samples), GSE53625 (179 paired specimens). The expression of ESCCAL-1 was consistently increased in ESCC tumors compared to normal tissues with the fold change from 4.5 to 25 (Fig. 1B–E). We further validated the expression pattern of ESCCAL-1 in additional 41 matched normal and tumor tissues from surgically excised specimens of ESCC by qRT-PCR. ESCCAL-1 was again significantly over-expressed in ESCC tumor tissues as compared to normal tissues (Fig. 1F). Moreover, the expression of ESCCAL-1 was also upregulated in 5 ESCC cell lines (Fig. 1G), which was consistent with its expression in tissues. We next analyzed the relationship between ESCCAL-1 expression and clinical characteristics of ESCC patients, and found that high expression of ESCCAL-1 was significantly correlated with poor tumor differentiation (*p* = 0.007) and slightly associated with advanced tumor stage (*p* = 0.072), but there is no positive correlation with age or gender (Supplementary Table 2). To investigate the potential clinical significance of ESCCAL-1 in ESCC diagnosis, we performed ROC analysis and found that the area under curve (AUC) of ESCCAL-1 in the 6 datasets (GSE120356, GSE53622, TCGA, GSE53624, GSE53625, and our cohort) were 1.0, 0.9378, 0.8264, 0.933, 0.9339, and 0.665 (Supplementary Fig. 1A–F), respectively. These data suggest that higher expression of ESCCAL-1 is a potentially promising diagnostic biomarker for ESCC.

### Overexpression of ESCCAL-1 enhances ESCC cell proliferation

**Fig. 2:**
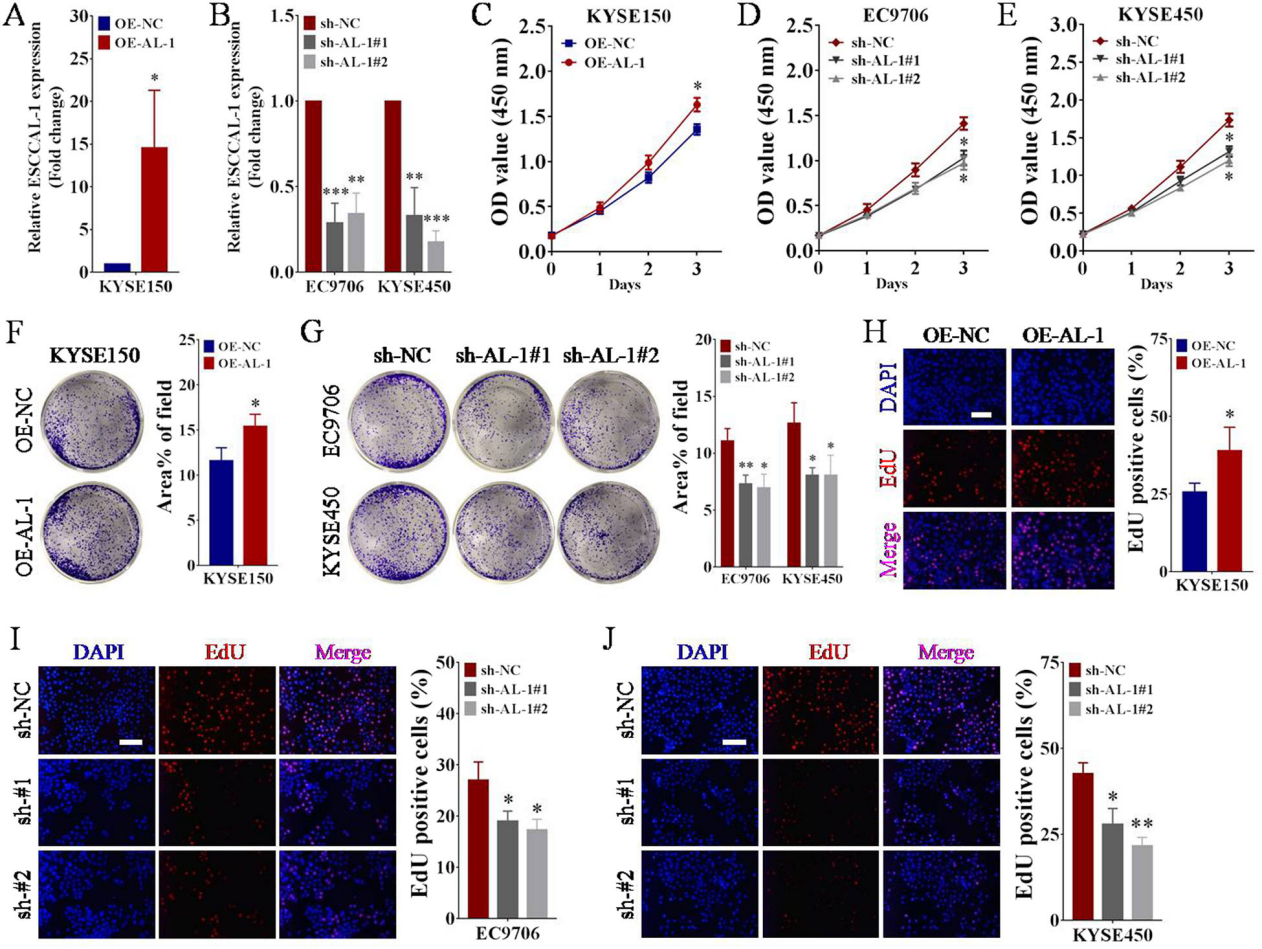
Overexpression of ESCCAL-1 enhances ESCC cell proliferation. **A**, **B** The relative expression level of ESCCAL-1 in ESCC cells following ESCCAL-1 overexpression (A) or two different shRNAs knockdown (**B**) was detected by qRT-PCR, *p < 0.05 as compared to OE-NC (**A**), **p < 0.01 or ***p < 0.001 as compared to sh-NC (**B**). **C**–**E** Cell growth of ESCC after ESCCAL-1 overexpression (**C**) or two different shRNAs knockdown (**D**, **E**) was examined with CCK-8 assay, *p < 0.05 as compared to OE-NC or sh-NC. **F**, **G** The population-independent growth ability of ESCC cells after ESCCAL-1 overexpression (**F**) or knockdown (**G**) was evaluated by colony formation assay, *p < 0.05 as compared to OE-NC (**F**), *p < 0.05 or **p < 0.01 as compared to sh-NC (**G**). **H**–**J** Cell proliferation of ESCC after ESCCAL-1 overexpression (**H**) or knockdown (**I**, **J**) was measured with EdU assay, *p < 0.05 as compared to OE-NC (**H**), *p < 0.05 or **p < 0.01 as compared to sh-NC (**I**, **J**). Scale bar = 100 μm.

We previously showed that knockdown of ESCCAL-1 reduced cell viability of ESCC in vitro^26,27^. To elucidate the oncogenic role of ESCCAL-1 in sustaining ESCC cell proliferation, we ectopically expressed ESCCAL-1 in a KYSE150 ESCC cell line, which the ESCCAL-1 expression is relative lower, and silenced ESCCAL-1 expression in EC9706 and KYSE450 ESCC cell lines with higher ESCCAL-1 expression, and performed CCK-8 experiment, colony formation assay, and EdU staining. The efficiencies of ESCCAL-1 overexpression (OE-AL-1) and knockdown (sh-AL-1#1, sh-AL-1#2) based on lentiviral recombinant vectors were confirmed by qRT-PCR (Fig. 2A, B). Overexpression of ESCCAL-1 increased the viability of KYSE150 cells (Fig. 2C), while knockdown of ESCCAL-1 decreased the viability of EC9706 and KYSE450 cells over time, significant differences were observed at day 3 (Fig. 2D, E). Colony formation assay indicated that overexpression of ESCCAL-1 enhanced the clonogenic ability of ESCC cells in vitro (Fig. 2F), while knockdown of ESCCAL-1 reduced colony formation (Fig. 2G). Furthermore, EdU incorporation assay revealed that ectopic expression of ESCCAL-1 facilitated DNA synthesis of KYSE150 cells (Fig. 2H), while downregulation of ESCCAL-1 suppressed DNA synthesis of EC9706 and KYSE450 (Fig. 2I, J). In addition, high expression of ESCCAL-1 promoted the migratory and invasive capacity of ESCC cells (Supplementary Fig. 2A), whereas ESCCAL-1 knockdown decreased cell migration and invasion (Supplementary Fig. 2B, C), which was consistent with our previous findings that depletion of ESCCAL-1 reduced the metastatic potential of ESCC cells^26,27^. Collectively, both gain-of-function and loss-of-function assays in vitro demonstrate that ESCCAL-1 promotes ESCC cell proliferation.

### Overexpression of ESCCAL-1 promotes ESCC cell-cycle progression

**Fig. 3:**
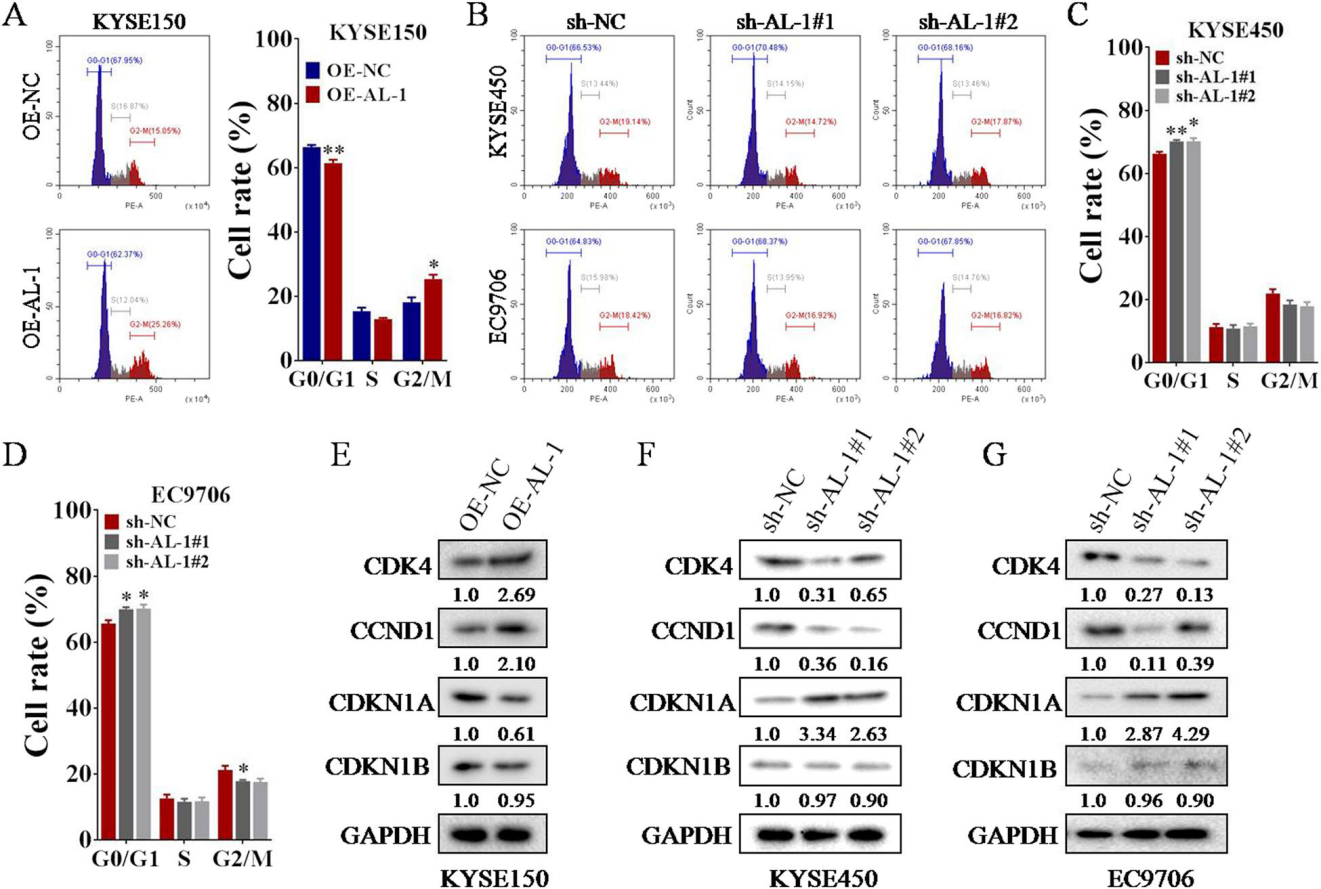
Overexpression of ESCCAL-1 promotes ESCC cell-cycle progression. **A**–**D** Flow cytometry was used to examine the effects of ESCCAL-1 overexpression or silencing with two shRNAs on cell cycle of ESCC. Significant cell cycle promotion and inhibition were observed when ESCCAL-1 was over-expressed (**A**) or knocked down (**B**–**D**), *p < 0.05 as compared to OE-NC or sh-NC. **E**–**G** The protein levels of cell-cycle regulators (CDK4, CCND1, CDKN1A, and CDKN1B) in ESCC cells following ESCCAL-1 overexpression (**E**) or knockdown (**F**, **G**) was tested by western blot. Representative western blot bands from two experiments were shown here. The intensities of expression bands were quantified, and the ratios relative to GAPDH were labeled under each blot.

Since cell cycle is one of the key biological processes related to cell proliferation^28,29^, we examined the effect of ectopic expression of ESCCAL-1 on ESCC cell cycle progress. Flow cytometry analysis indicated that overexpression of ESCCAL-1 led to a decrease of G0/G1 phase and an increase of G2/M phase in KYSE150 expressing ESCCAL-1 relative to the control (Fig. 3A), while two independent shRNAs-mediated knockdown of ESCCAL-1 elicited the opposite results (Fig. 3B–D). Since the CDK4-Cyclin D1 (*CCND1*) complexes and cyclin-dependent kinase inhibitors (CKIs) CDKN1A and CDKN1B are key regulators in controlling cell-cycle progression, we then detected their protein levels in ESCC cells following ESCCAL-1 overexpression or knockdown by western blot. The results showed that high expression of ESCCAL-1 upregulated the protein levels of CDK4 and CCND1 by more than 2-folds versus controls (Fig. 3E), while knockdown of ESCCAL-1 decreased their protein levels by up to 70% (Fig. 3F, G). Intriguingly, overexpression of ESCCAL-1 decreased the protein level of CDKN1A by ~40% but not CDKN1B (Fig. 3E), and knockdown of ESCCAL-1 remarkably increased CDKN1A protein levels by at least 2.5-folds (Fig. 3F, G). We also examined the changes of CDK4, CCND1, CDKN1A, and CDKN1B at transcriptional level in ESCC cells following ESCCAL-1 knockdown or overexpression. The results showed that the mRNA levels of CDK4, CCND1, CDKN1A, and CDKN1B did not change significantly in ESCC cells after knockdown or overexpression of ESCCAL-1 (Supplementary Fig. 3A, B). Moreover, we performed rescue experiment and found that overexpression of CDK4 significantly relieved ESCC cell-cycle arrest induced by ESCCAL-1 knockdown (Supplementary Fig. 3C). These results suggest that CDK4, CCND1, and CDKN1A contribute to ESCCAL-1-mediated ESCC cell-cycle progression.

### LncRNA ESCCAL-1 directly binds to Gal-1 protein and positively regulates its protein level

**Fig. 4:**
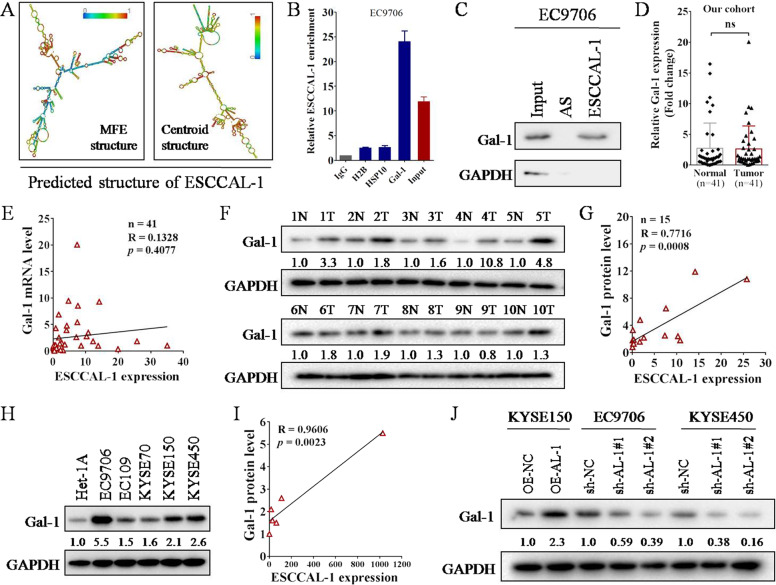
LncRNA ESCCAL-1 interacts with oncoprotein Gal-1 and positively regulates its protein level. **A** The secondary structure of ESCCAL-1 transcript in minimum free energy (MFE) and Centroid modes was predicted by the online tool RNAfold. **B** The direct binding effects between ESCCAL-1 transcript and three candidate proteins (H2B, HSP10, and Gal-1) were detected by RNA immunoprecipitation (RIP). RNA-containing eluents from the RIP experiment were used for qRT-PCR detection. **C** Gal-1 was identified as an ESCCAL-1 bound substrate in ESCC cells by RNA-protein pull-down assay. Antisense (AS) of ESCCAL-1 RNA was used as negative control. **D** The RNA expression of Gal-1 was determined in 41 pairs of ESCC tissues by qRT-PCR. NS means no significance. **E** Pearson coefficient was used to analyze the correlation between ESCCAL-1 expression and Gal-1 mRNA level in 41 cases of ESCC tissues. **F** The protein levels of Gal-1 in 15 paired ESCC tumor tissues (T) and matched adjacent normal tissues (N) were assessed by western blot (showing 10 pairs). **G** Pearson coefficient was used to analyze the correlation between ESCCAL-1 expression and Gal-1 protein level in 15 cases of ESCC tissues. **H** The protein levels of Gal-1 in 5 ESCC cell lines (EC9706, EC109, KYSE70, KYSE150, and KYSE450) and a normal epithelial cell line (Het-1A) were determined by western blot. **I** Pearson coefficient was used to analyze the correlation between ESCCAL-1 expression and Gal-1 protein level in 6 cell lines (Het-1A, EC9706, EC109, KYSE70, KYSE150, and KYSE450). **J** The protein levels of Gal-1 in ESCC cells after ESCCAL-1 overexpression or knockdown were tested by western blot. The intensity of Gal-1 expression was quantified and labeled under each blot.

LncRNAs can fulfill their specific functions by interacting with various biological molecules such as DNA, microRNAs, and proteins, which is closely related to its secondary structure^30,31^. The secondary structure of ESCCAL-1 transcript in minimum free energy (MFE) and Centroid modes was analyzed by the online tool *RNAfold*, both predicted a Y-shaped structure (Fig. 4A), suggesting that ESCCAL-1 harbors protein binding potential. Indeed, we identified a panel of proteins that may physically interact with ESCCAL-1 using RNA-protein pull-down followed by mass spectrometry analysis (Supplementary Table 3). We then selected the top-3 ESCCAL-1 bound proteins with the highest coverage reliability, HSP10, Gal-1, and H2B, for RIP verification. Gal-1 was confirmed as a substrate protein for lncRNA ESCCAL-1 binding as shown in Fig. 4B, C. In addition, we performed immunofluorescence staining to detect the co-localization of ESCCAL-1 RNA and Gal-1 protein in ESCC cells. The result showed that ESCCAL-1 and Gal-1 protein co-located in KYSE150 cells (Supplementary Fig. 4A), further indicating the intracellular interaction between ESCCAL-1 and Gal-1 protein.

To investigate the expression pattern of Gal-1, we detected its transcription in collected 41 pairs of fresh surgical specimens of ESCC by qRT-PCR, we observed there is no significant difference in the mRNA levels of Gal-1 between tumor and normal tissues (Fig. 4D). In addition, there was no significant correlation between the mRNA level of Gal-1 and the transcription level of ESCCAL-1 in 41 cases of ESCC specimens (Fig. 4E). Data from GEPIA database were consistent with our results (Supplementary Fig. 4B, C). However, the protein levels of Gal-1 were obviously upregulated in 12 out of 15 cases of ESCC tumor tissues as compared to normal tissues (Fig. 4F, Supplementary Fig. 4D). The protein levels of Gal-1 were positively correlated with the transcription levels of ESCCAL-1 in 15 cases of ESCC tumor tissues (Fig. 4G). Furthermore, we also found that the protein level of Gal-1 in ESCC cell lines was upregulated and positively correlated with the expression of ESCCAL-1 (Fig. 4H, I). In addition, overexpression of ESCCAL-1 increased the protein level of Gal-1 in ESCC cells, while knockdown of ESCCAL-1 downregulated the protein level of Gal-1 (Fig. 4J). But ESCCAL-1 manipulation had no significant effect on the mRNA level of Gal-1 (Supplementary Fig. 4E). Gal-1 manipulation had no significant effect on ESCCAL-1 expression (Supplementary Fig. 4F). These results indicate that lncRNA ESCCAL-1 directly binds to Gal-1 protein and positively regulates its protein level, and Gal-1 protein is the downstream molecule of ESCCAL-1.

### Gal-1 functions as an oncogenic protein that promotes ESCC cell proliferation and cell-cycle progression

**Fig. 5:**
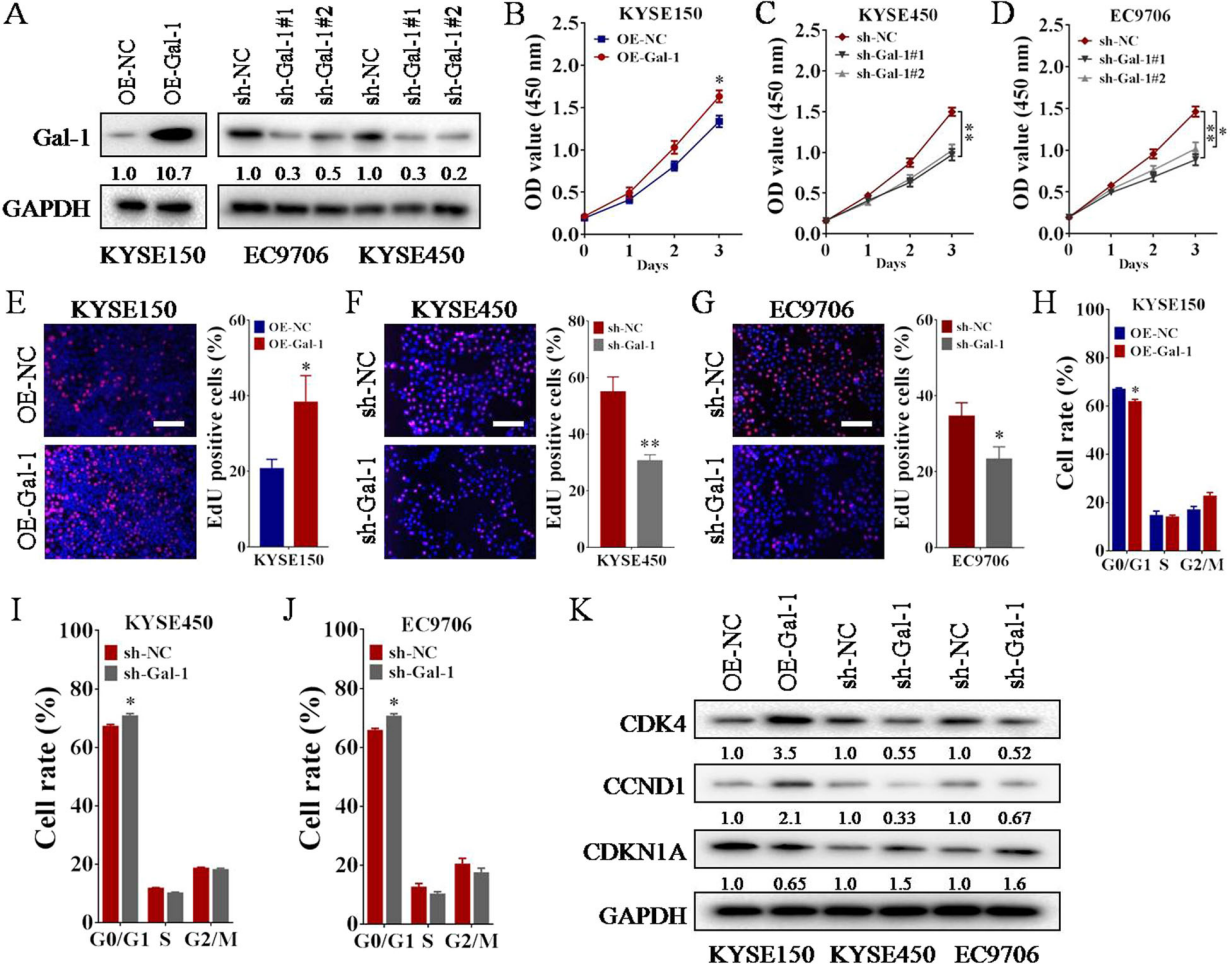
Overexpression of Gal-1 facilitates ESCC cell proliferation. **A** The protein levels of Gal-1 in ESCC cells following Gal-1 overexpression (OE-Gal-1) or knockdown (sh-Gal-1#1, sh-Gal-1#2) were determined by western blot. **B**–**D** Cell proliferation of ESCC after Gal-1 overexpression (**B**) or knockdown (**C**, **D**, sh-Gal-1#1 and sh-Gal-1#2) was tested with CCK-8 assay, *p < 0.05 or **p < 0.01 as compared to OE-NC or sh-NC. **E**–**G** Cell proliferation of ESCC after Gal-1 overexpression (**E**) or knockdown (**F**, **G**, sh-Gal-1#1) was examined with EdU assay, *p < 0.05 as compared to OE-NC (**E**), *p < 0.05 or **p < 0.01 as compared to sh-NC (**F**, **G**). Scale bar = 100 μm. **H**–**J** Flow cytometry was performed to examine the effects of Gal-1 manipulation on cell cycle of ESCC. Significant cell cycle promotion and inhibition were observed when Gal-1 was over-expressed (H) or knocked down (**I**, **J**, sh-Gal-1#1), *p < 0.05 as compared to OE-NC or sh-NC. **K** The protein levels of cell-cycle regulators (CDK4, CCND1, and CDKN1A) in ESCC cells following Gal-1 overexpression or knockdown (sh-Gal-1#1) was tested by western blot.

To characterize the biological role of Gal-1 in cell proliferation of ESCC, we performed CCK-8 experiment and EdU incorporation staining. The efficiencies of Gal-1 overexpression (OE-Gal-1) and knockdown (sh-Gal-1#1 and sh-Gal-1#2) based on lentiviral recombinant vectors were determined by western blot (Fig. 5A). Results of both CCK-8 test and EdU staining showed that overexpression of Gal-1 increased cell proliferation of KYSE150 (Fig. 5B, E), while knockdown of Gal-1 decreased the proliferative ability of KYSE450 and EC9706 (Fig. 5C, D, F, G). Subsequently, we examined the effect of ectopic expression of Gal-1 on ESCC cell cycle progression. Flow cytometry analysis demonstrated that overexpression of Gal-1 led to a significant decrease of G0/G1 phase (Fig. 5H, Supplementary Fig. 5A), while knockdown of Gal-1 increase G0/G1 phase of ESCC cell cycle (Fig. 5I, J, Supplementary Fig. 5B). In addition, western blot revealed that high expression of Gal-1 upregulated the protein levels of CDK4 and CCND1 by at least 2-folds when compared to controls (Fig. 5K), while knockdown of Gal-1 downregulated their protein levels by up to 60% (Fig. 5K). Overexpression of Gal-1 decreased the protein level of CDKN1A (Fig. 5K) but not CDKN1B (Supplementary Fig. 5C), and knockdown of Gal-1 was consistent with this result (Fig. 5K). Collectively, both gain-of-function and loss-of-function assays indicate that Gal-1 promotes ESCC cell proliferation and cell-cycle progression.

### ESCCAL-1 promotes ESCC cell-cycle progression via Gal-1

**Fig. 6:**
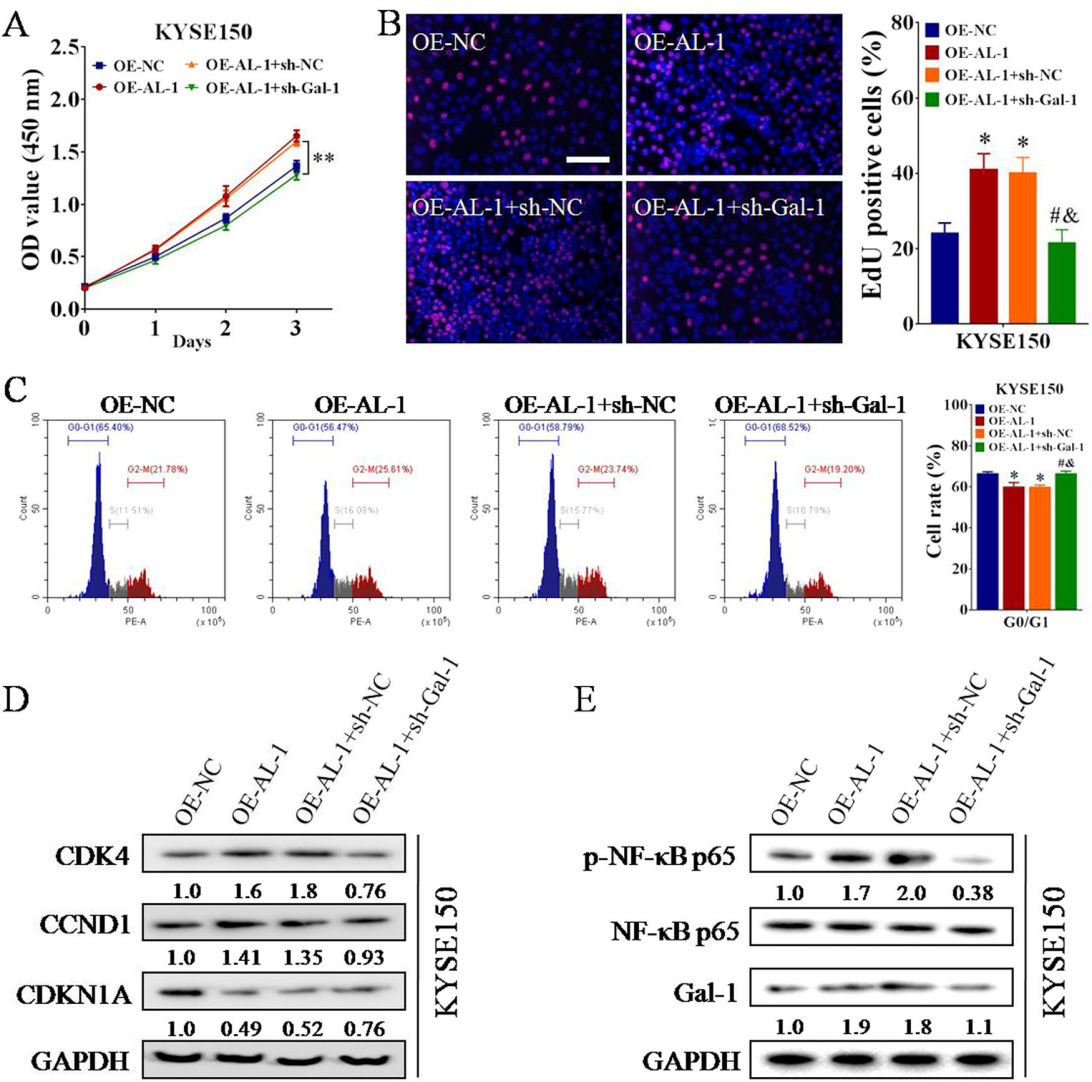
ESCCAL-1 promotes ESCC cell proliferation and cycle via Gal-1. **A**, **B** Functional recovery assays of CCK-8 (**A**) and EdU (**B**) revealed that the effects of ESCCAL-1 overexpression on ESCC cell proliferation were significantly abolished by Gal-1 knockdown. For **A**, **p < 0.01 as compared to OE-AL-1+sh-NC; for **B**, *p < 0.05 as compared to OE-NC, ^#^*p* < 0.05 as compared to OE-AL-1, and ^&^*p* < 0.05 as compared to OE-AL-1+sh-NC. Scale bar = 100 μm. **C** Flow cytometry was performed to examine the effects of ESCCAL-1-Gal-1 interaction on cell cycle of ESCC, *p < 0.05 as compared to OE-NC, ^#^*p* < 0.05 as compared to OE-AL-1, ^&^*p* < 0.05 as compared to OE-AL-1+sh-NC. **D, E** The protein levels of cell-cycle regulators (**D**; CDK4, CCND1, and CDKN1A) and NF-κB signaling (**E**; p65 and p-p65) in ESCC cells were tested by western blot.

Considering that ESCCAL-1 directly binds to Gal-1 protein, and they have similar biological functions in regulating ESCC cell proliferation and cycle, we hypothesized that ESCCAL-1 plays an oncogenic role in ESCC through the interaction with Gal-1. To test this hypothesis, we performed rescue experiments and measured the biological functions using both CCK-8 test and EdU staining. Depletion of Gal-1 significantly impaired OE-ESCCAL-1-induced high cell proliferation in ESCC (Fig. 6A, B). Meanwhile, flow cytometry and western blot analysis revealed that the effects of ESCCAL-1 overexpression on cell cycle and cell cycle regulators (CDK4, CCND1, and CDKN1A) were abolished, at least in part, by knockdown of Gal-1 in ESCC cells (Fig. 6C, D). To explore the possible signaling pathways mediated by Gal-1, we used the online tool STRING to predict a PPI network of Gal-1 protein. In this network, as one of the signaling molecules most closely associated with Gal-1 protein, NF-κB has attracted our attention (Supplementary Fig. 6), because numerous previous studies have elucidated that the abnormal activation of NF-κB signaling is related to tumor progression, including ESCC^32,33^. Western blot showed that overexpression of ESCCAL-1 resulted in activation of NF-κB, and this signal was significantly reduced when Gal-1 was simultaneously knocked down (Fig. 6E). These results suggest that ESCCAL-1 promotes ESCC cell-cycle progression may be through Gal-1-dependent NF-κB activation.

### ESCCAL-1 promotes ESCC tumor growth in a Gal-1-mediated manner

**Fig. 7:**
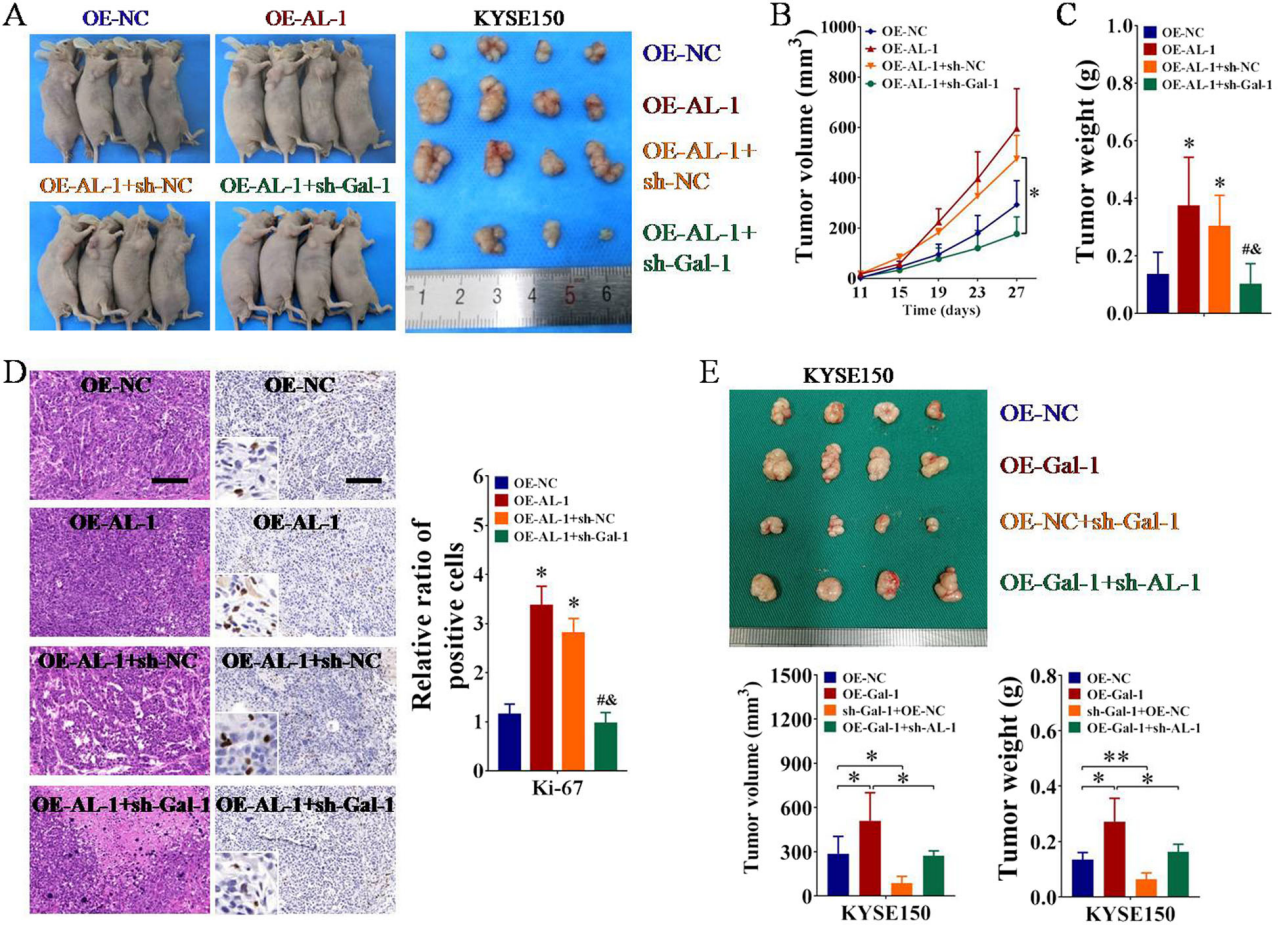
ESCCAL-1 promotes tumorigenesis of ESCC in a Gal-1-dependent manner. **A**–**C** Tumor formation experiment in nude mice in vivo was performed to examine the effects of ESCCAL-1-Gal-1 interaction on tumorigenesis of ESCC. Knockdown of Gal-1 alleviated the promotion of ESCCAL-1 overexpression on ESCC tumorigenesis (**A**), as indicated by tumor volume (**B**) and tumor weight (**C**). For **B**, *p < 0.05 as compared to OE-AL-1+sh-NC; for **C**, *p < 0.05 as compared to OE-NC, ^#^*p* < 0.05 as compared to OE-AL-1, and ^&^*p* < 0.05 as compared to OE-AL-1+sh-NC. **D** The tissue sections from the xenografts tumors were stained with hematoxylin and eosin (HE). The levels of Ki-67 were tested by immunohistochemistry (IHC), *p < 0.05 as compared to OE-NC, ^#^*p* < 0.05 as compared to OE-AL-1, and ^&^*p* < 0.05 as compared to OE-AL-1+sh-NC. Scale bar = 100 μm. **E** Further in vivo rescue experiment in nude mice was conducted to validate the effects of ESCCAL-1/Gal-1 axis on ESCC tumor growth, *p < 0.05 or **p < 0.01.

To further elucidate the involvement of ESCCAL-1-Gal-1 interaction in controlling ESCC tumorigenesis, tumor formation experiment in nude mice was conducted. The results showed that tumors derived from ESCCAL-1 over-expressed cells grew much faster, as indicated by larger tumor size and heavier tumor weight (Fig. 7A–C). However, depletion of Gal-1 significantly impaired the promotion of ESCCAL-1 overexpression on ESCC tumor growth (Fig. 7A–C). The level of proliferation marker Ki-67 in xenograft tumors was then detected by IHC staining. The results displayed that the number of Ki-67-positive cells in ESCCAL-1 overexpression group (OE-AL-1) was significantly increased when compared to control group (OE-NC), which further indicated that overexpression of ESCCAL-1 promoted the proliferation of ESCC cells in vivo (Fig. 7D). But the increase of Ki-67-positive cells induced by ESCCAL-1 overexpression was obviously offset by Gal-1 knockdown (Fig. 7D). Moreover, consistent with the in vitro data (Fig. 6D), western blot analysis revealed that the effects of ESCCAL-1 overexpression on cell cycle regulators (CDK4, CCND1, and CDKN1A) were partially abolished by knockdown of Gal-1 in ESCC tumor tissues (Supplementary Fig. 7).

Next, we performed in vivo rescue experiments again to confirm the role of ESCCAL-1/Gal-1 axis in ESCC growth. As shown in Fig. 7E, we found that overexpression of Gal-1 promoted tumor growth, while knockdown of Gal-1 inhibited tumor growth in vivo, further indicating the oncogenic role of Gal-1 in ESCC. Moreover, ESCCAL-1 knockdown could significantly relieve the tumor-promoting effect caused by OE-Gal-1, demonstrating the involvement of ESCCAL-1/Gal-1 axis in the progression of ESCC. Together, these data suggest that ESCCAL-1 promotes ESCC tumor growth in a Gal-1-dependent manner.

### ESCCAL-1 enhances Gal-1 protein stability by blocking Smurf1-mediated ubiquitination

**Fig. 8:**
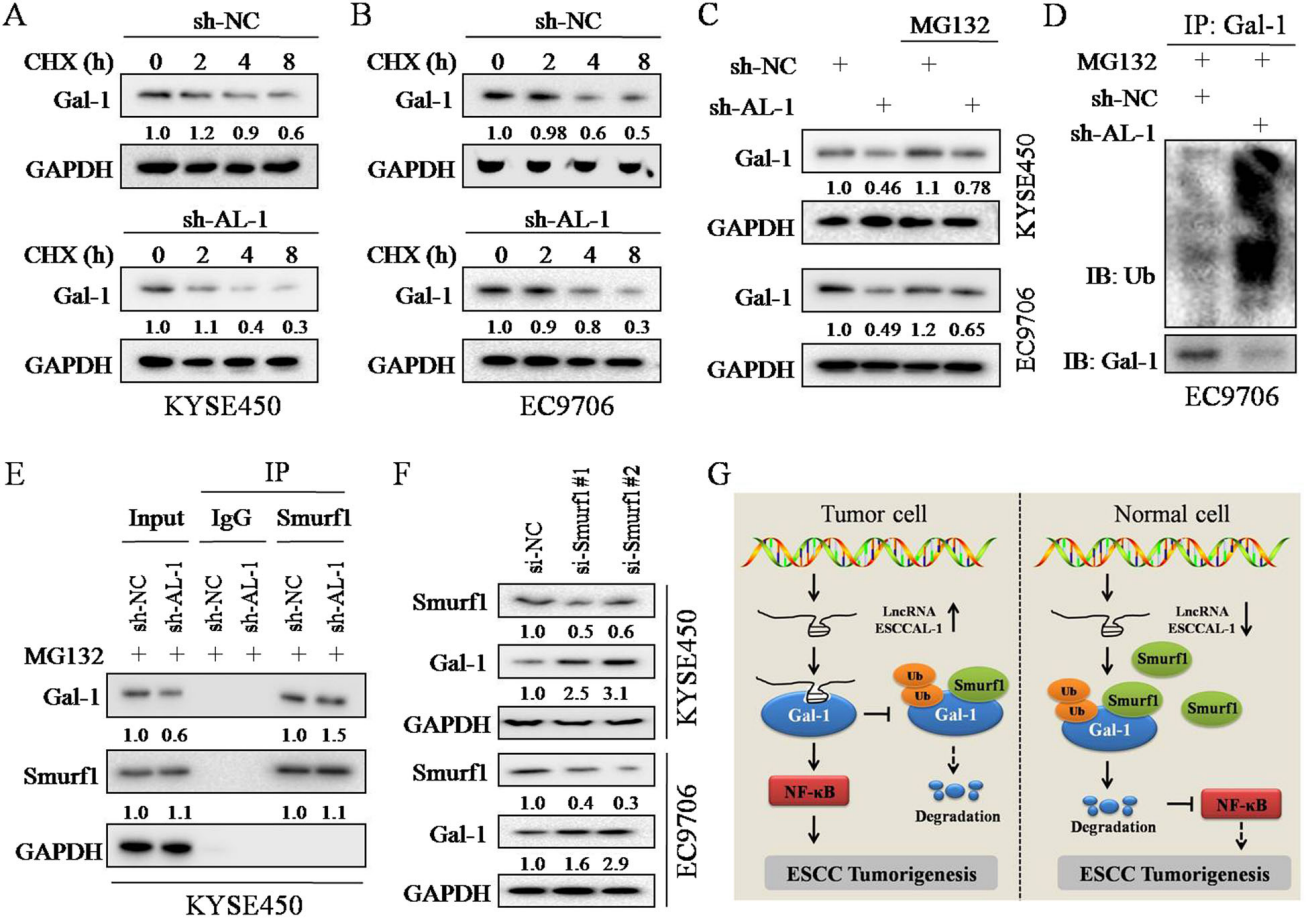
ESCCAL-1 stabilizes Gal-1 protein by blocking Smurf1-mediated ubiquitination. **A**, **B** The protein levels of Gal-1 in ESCC cells treated with cycloheximide (CHX, concentration at 1 μg per mL) for the indicated time were tested by western blot. Knockdown of ESCCAL-1 (sh-AL-1) shortened the half-life of Gal-1 protein in ESCC cells. **C** The proteasome inhibitor MG132 (5 μM) partially abolished the decrease in Gal-1 protein level caused by ESCCAL-1 knockdown. **D** Co-immunoprecipitation (Co-IP) combined with western blot revealed that ESCCAL-1 knockdown promoted Gal-1 ubiquitination. Protein-containing eluents from the IP assay (anti-Gal-1) were used for western blot detection. **E** Co-IP combined with western blot revealed that ESCCAL-1 knockdown promoted the interaction between Smurf1 and Gal-1 protein. **F** The protein levels of Gal-1 in ESCC cells after knockdown of E3 ubiquitin ligase Smurf1 were examined by western blot. **G** The working model of ESCCAL-1 promotes ESCC tumorigenesis via interacting with and stabilizing Gal-1.

Given that ESCCAL-1 directly interacts with Gal-1 protein and positively associates with Gal-1 protein level with no impingement on Gal-1 mRNA level, we intend to explore the potential mechanism that is responsible for ESCCAL-1-mediated increase of Gal-1 protein in ESCC cells. First, we found that knockdown of ESCCAL-1 shortened the half-life time of Gal-1 protein when treated with cycloheximide (CHX) for the indicated times in KYSE450 and EC9706 cells (Fig. 8A, B). In addition, the reduction of Gal-1 protein induced by ESCCAL-1 knockdown was abolished, at least in part, by the proteasome inhibitor MG132 (Fig. 8C), suggesting that ESCCAL-1 protects Gal-1 protein through blocking its ubiquitination-mediated degradation. Further IP combined with western blot confirmed that knockdown of ESCCAL-1 significantly promoted ubiquitination of Gal-1 in ESCC cells in the presence of MG132 (Fig. 8D), suggesting that ESCCAL-1 stabilizes Gal-1 protein by preventing its degradation through ubiquitin-proteasome pathway (UPP).

To preliminarily investigate the underlying mechanism by which ESCCAL-1 depletion leads to Gal-1 degradation through UPP, we searched for E3 ubiquitin ligases that may mediate Gal-1 ubiquitination modification. We focused on two candidate molecules, Smurf1 and CUL4A for experimental validation. Because Smurf1 is the most reliable E3 ubiquitin ligase that mediates the ubiquitination modification of Gal-1 protein predicted by bioinformatics tool UbiBrowser (Supplementary Fig. 8A), CUL4A, a member of many E3 ligase complexes, has been reported in a previous study to play an important role in the progression of ESCC^34^. Further IP combined with western blot revealed that knockdown of ESCCAL-1 promoted the binding of Smurf1 to Gal-1 protein (Fig. 8E), but not CUL4A (Supplementary Fig. 8B), indicating that Gal-1 protein is one of the substrates of Smurf1 rather than CUL4A. In addition, we found that siRNA-mediated ablation of Smurf1 increased the protein level of Gal-1 in ESCC cells (Fig. 8F). We then performed rescued experiments and found that knockdown or overexpression of Smurf1 removed the ESCCAL-1-dependent changes in Gal-1 protein level (Supplementary Fig. 9), suggesting that ESCCAL-1 stabilizes Gal-1 protein by blocking Smurf1-mediated ubiquitination. These data suggest that ESCCAL-1 stabilizes Gal-1 protein by blocking Smurf1-mediated ubiquitination (Fig. 8G).

## Discussion

LncRNAs are widely expressed in mammalian genome. They play crucial roles in gene regulation and tumorigenesis, and participate in biological processes through specific interactions with biomolecules^21,35^. LncRNAs express in tissue-specific expression pattern and act as potential biomarkers and therapeutic targets. Targeting lncRNAs is becoming a new approach for human tumor therapy^20,21,35^. Our previous studies demonstrated that depletion of ESCCAL-1 expression inhibits the growth of ESCC cells^27,36^, we report here a novel mechanism whereby ESCCAL-1 promotes cell-cycle progression via binding and stabilizing Gal-1 protein through blocking its ubiquitination.

Several drugs have been successfully applied in clinical practice to combat malignant proliferation of tumors by targeting inhibition of CCND1-CDK4 and other cell cycle regulators^37^. However, cancer cells resistant to these CDK4 inhibitors still rely on CCND1 for proliferation^38,39^, suggesting that new strategies to suppress CCND1-CDK4 are urgently needed. In this study, our results showed that the oncogenic lncRNA ESCCAL-1 promoted ESCC cell-cycle progression, upregulated the protein levels of CDK4 and CCND1, and downregulated CDKN1A, indicating that ESCCAL-1 is a potential molecular therapy target for ESCC. More importantly, in order to explore the diagnostic utility of ESCCAL-1, we analyzed its expression profile in multiple ESCC cohorts, including our own cohort. The prediction probability of ESCCAL-1 as a biomarker of ESCC is between 0.665 to 0.937 in six different cohorts. A recent study examined the level of a 4-lncRNA panel (including ESCCAL-1) in ESCC serum-derived exosomes and evaluated its diagnostic value, indicating that this panel was significantly more specific than squamous cell carcinoma antigen^40^. In line with this, our finding suggested that ESCCAL-1 could become a promising clinical biomarker for ESCC. The reliability of ESCCAL-1 as an ESCC biomarker based on blood test requires further investigation with a larger sample size.

Emerging evidence highlights that the oncogenic role of Gal-1 depends on its imbalanced expression. The use of inhibitors to suppress Gal-1 is expected to be an effective clinical treatment strategy for cancer^41–43^. Previous studies have shown that Gal-1 is implicated in regulating cancer cell proliferation^44,45^ and may play a role in ESCC development^19^. However, the specific expression pattern and role of Gal-1 in ESCC remain elusive. In this study, Gal-1 was over-expressed in ESCC as indicated by its protein level rather than its transcription level, implying that Gal-1 may be regulated by post-translational modifications and thus plays a biological role in ESCC. We identified Gal-1 directly binds to ESCCAL-1 in ESCC cells using RNA-protein pull-down, RIP, and immunofluorescence co-location assays, to our knowledge, it is the first time to report that Gal-1 can act as an RNA-binding protein in tumor cells, the detailed protein binding motif in ESCCLA-1 remain further characterized in the future study. Subsequent in vitro and in vivo functional rescue experiments demonstrated that ESCCAL-1 promotes cell-cycle progression and tumorigenesis of ESCC through Gal-1.

E3 ubiquitin ligase can maintain protein balance and cell homeostasis by mediating the ubiquitination modification of functional proteins. Once the ubiquitination regulation of key proteins is abnormal, cells with persistent imbalance in homeostasis may become cancerous^46,47^. As one of the more active E3 ubiquitin ligases, Smurf1 has been reported to be involved in tumor progression. For instance, Smurf1 accelerates PTEN ubiquitination and thus mediates prostate cancer and glioblastoma progression through the mTOR signaling^48,49^. Although the precise mechanism is unascertained, Smurf1 ablation affects the sensitivity of colorectal cancer to chemotherapeutic agents such as gemcitabine^50^. However, it is not clear whether Smurf1 plays a role in ESCC. In this study, we uncovered that knockdown of ESCCAL-1 promotes ubiquitination degradation of Gal-1 protein in Smurf1-dependent manner in ESCC cells, suggesting that Smurf1 may be one of the key E3 ligases regulating ESCC development.

We have shown that ESCCAL-1 plays an oncogenic role in ESCC through Gal-1. NF-κB appears to be a downstream effector following Gal-1 overexpression in epithelial ovarian cancer^51^. We displayed that the effect of ESCCAL-1 overexpression on NF-κB activation was attenuated by Gal-1 depletion, suggesting that ESCCAL-1/Gal-1 promotes ESCC could be through NF-κB activation. However, it remains unclear how the ESCCAL-1-Gal-1 axis activates NF-κB in ESCC, which needs to be further explored and validated.

In conclusion, we demonstrated that overexpression of ESCCAL-1 promoted ESCC progression by modulating the cell-cycle regulators. LncRNA ESCCAL-1 physically interacts with Gal-1 protein and prevents the protein complex formation of E3 ubiquitin ligase Smurf1 and Gal-1, resulting in stabilization of Gal-1 protein. This novel mechanism of lncRNA-protein interaction provides potential therapeutic strategies for ESCC.

## Materials and methods

### Tissue specimens

A total of 41 pairs of ESCC tumor tissues and matched adjacent normal tissues were collected from the Linzhou Cancer Hospital. All patients did not receive any chemoradiotherapy prior to surgery and signed written informed consent. All fresh specimens were immediately frozen at liquid nitrogen and kept in an ultra-low temperature refrigerator for a long time. The experimental procedures were approved by the Ethics Committee of the Zhengzhou Central Hospital Affiliated to Zhengzhou University.

### Cell culture and cell transfection

Two ESCC cell lines EC9706 and EC109 were previously obtained from the Cell Bank of Shanghai Academy of Biological Sciences. One normal esophagus epithelial cell line Het-1A and three ESCC cell lines KYSE70, KYSE150 and KYSE450 were kindly provided by the Bioengineering and Transformation Laboratory of Zhengzhou University. Each cell line abovementioned was grown in RPMI 1640 medium supplemented with 10% fetal bovine serum (FBS) (Gibco, USA) and maintained in a cell incubator at 37 °C containing 5% CO_2_. Lentiviral vectors for gene knockdown (sh-ESCCAL-1#1 and sh-ESCCAL-1#2, sh-Gal-1#1 and sh-Gal-1#2) or overexpression (OE-ESCCAL-1, OE-Gal-1, OE-CDK4, OE-Smurf1) as well as the corresponding negative control vectors (sh-NC, OE-NC) were purchased from Shanghai GeneChem Company. The siRNAs used for Smurf1 knockdown (si-Smurf1#1 and si-Smurf1#2) were synthesized from Shanghai GenePharma Company and the sequence is shown in Supplementary Table 1. Infection enhancer HitransG A (Shanghai Genechem, China), Lipofectamine 3000 (Invitrogen, USA), and transfection reagent INTERFERin (Polyplus, France) were employed to transfect lentiviral vectors, plasmids, and siRNAs into ESCC cells, respectively.

### Quantitative real-time polymerase chain reaction (qRT-PCR)

Total RNA from cells or tissues after grinding was extracted by Trizol (Invitrogen, USA) method. After quality evaluation of the RNA samples and first-strand cDNA synthesis (Novoprotein, China), qRT-PCR was performed to detect gene expression using SYBRGreen PCR kit (DBI, Germany) and the 7500 Fast Real-Time PCR System (Applied Biosystems, USA) according to the user’s manual. The house-keeping gene GAPDH was used as internal control, and the transcription levels of ESCCAL-1 and Gal-1 were presented as fold change using 2^−ΔΔCt^ method. The primers used in this study are listed in Supplementary Table 1.

### Cell proliferative assays

For cell counting kit 8 (CCK-8) analysis, ESCC cells were seeded into 96-well plates (5 × 10^3^ cells per well). At the indicated time points, 10 μL of CCK-8 solution (7Sea Biotech, China) was added to each well and incubated at 37 °C for 4 h. Then the absorbance of each well at 450 nm was detected using a microplate reader (Molecular Devices, USA). For colony formation assay, ESCC cells were seeded into 24-well plates (1000 cells per well) and maintained in a cell incubator for 7–10 days. Finally, the colonies were fixed with methanol and stained with crystal violet, and counted by Image J software. For 5-ethynyl-2’-deoxyuridine (EdU) assay, ESCC cells were marked with EdU solution (Abbkine, USA) at 37 °C for 4 h. Then, the cells were fixed with 4% formaldehyde and permeated with 0.5% TritonX-100, and reacted in Click-iT mixture for 30 min under dark. Finally, cells were stained with DAPI solution and photographed under an inverted fluorescence microscope.

### Flow cytometry for cell cycle

ESCC cells were fixed with 70% ethanol at 4 °C for 2 h, and subsequently reacted in staining buffer containing PI (7Sea Biotech, China) and RNase at 37 °C for 30 min. Cell cycle was finally tested by a Flow Cytometer (Beckman, USA).

### Transwell assays

Cell migration and invasion were examined by Transwell assays (Chamber with 8 μm membrane, Corning, USA). Briefly, cells in serum-free medium were seeded into the upper chamber coated without or with Matrigel. The lower chamber contained 500 μL culture medium with 20% FBS. After incubation at 37 °C for 24 h, the migratory or invasive cells on the surface of the lower chamber were fixed with formaldehyde and stained with crystal violet, and finally photographed under an invert microscope.

### Western blot analysis

Total proteins were extracted from tissues or cells using RIPA lysis buffer (containing 1% PMSF) and then denatured at 100 °C using a Heating Block (Hangzhou Bioer Technology, China). Equal amounts of proteins were separated by SDS-PAGE and then transferred onto PVDF membrane. After blocking with 5% skim milk and incubation with primary antibody and secondary antibody, the immunoreactive bands on the membrane were reacted with ECL solution and detected by the Chemidoc EQ system (BioRad, USA). Primary antibodies used in this study were as follows: anti-GAPDH (1:5000, Bioworld, China), anti-CCND1 (1:2000, Bioworld, China), anti-CDK4 (1:2000, Bioworld, China), anti-CDKN1A (1:500, Bioworld, China), anti-CDKN1B (1:1000, Bioworld, China), anti-NF-κB p65 (1:2000, Abcam, USA), anti-p-NF-κB p65 (1:2000, Abcam, USA), anti-Galectin-1 (1:500, Santa Cruz, USA), anti-Ubiquitin (1:200, Santa Cruz, USA), anti-Smurf1 (1:500, Santa Cruz, USA), anti-CUL4A (1:1000, Abnova, China). For each experiment, the blots were from the same experiment and were processed in parallel.

### RNA-protein pull-down assay

The procedures were following the user’s instructions of the Pierce 3’-End Desthiobiotinylation Kit (Thermo, USA) and the Magnetic RNA-Protein Pull-Down Kit (Thermo, USA). The PCR products of ESCCAL-1 were purified and then cloned into pGEM-T vector (Promega, USA) containing T7 promoter. The recombinant vector of ESCCAL-1-pGEM-T was used as template to obtain ESCCAL-1 transcripts by utilizing the Ultra-high Yield In Vitro Transcription Kit (Thermo, USA). The ESCCAL-1 transcripts were labeled with desthiobiotin and then incubated with streptavidin magnetic beads for 30 min at room temperature with agitation in a clean tube. Total proteins extracted from EC9706 cells were added to the tube and incubated at 4 °C for 1 h with rotation to generate the RNA-Protein complexes. After washing and eluting, the RNA-bound proteins were collected and used for mass spectrometric analysis and western blot verification.

### RNA-binding protein immunoprecipitation (RIP)

The EZ-Magna RIP Kit (Millipore, USA) was used to perform the RIP experiment according to manufacturer’s instructions. Briefly, EC9706 cells were lysed in RIP lysis buffer containing protease inhibitor cocktail and RNase inhibitor. After preparation of magnetic beads in the tube, 5 μg of antibodies (normal rabbit IgG as negative control; anti-Galectin-1; anti-H2B; anti-HSP10) were added to each tube and incubated for 30 min at room temperature. Then, 100 μL of cell lysates were added to each tube containing antibody-bead complexes and incubated at 4 °C overnight. After washing, proteinase K was added to each tube and incubated at 55 °C for 30 min to digest the proteins. Finally, RNAs were purified with phenol/chloroform/isoamyl alcohol extraction followed by ethanol precipitation. RNAs obtained from RIP were analyzed by qRT-PCR.

### Immunoprecipitation (IP)

The Immunoprecipitation assay was performed using the Pierce Classic Magnetic IP Kit (Thermo, USA). Briefly, total proteins isolated from cells were incubated with the diluted primary antibodies overnight at 4 °C. After that, pre-washed protein A/G magnetic beads were added to each sample and incubated at room temperature for 1 h. The beads were collected using a magnetic stand, and elution buffer was added to each sample to magnetically separate the beads and save the supernatant. Finally, the supernatant containing the target antigen was used for western blot analysis.

### Xenografts in nude mice

ESCC cells suspended in normal saline were inoculated subcutaneously on the right dorsal side of 6-week-old BALB/c nude mice (8 × 10^5^ cells per nude mice for Fig. 7A and 1.2 × 10^6^ cells per mice for Fig. 7E). The tumor size was measured using a length × width^2^ × 0.5 formula. Finally, the mice were sacrificed and the isolated tumors were weighed. Some tumor specimens were frozen at −80 °C for qRT-PCR and western blot analysis, and some were fixed with 4% paraformaldehyde for hematoxylin-eosin (HE) staining and Ki-67 immunohistochemical staining. The animal study was approved by the Ethics Committee of the Zhengzhou Central Hospital Affiliated to Zhengzhou University.

### Immunofluorescence co-location assay

The immunofluorescence assay was performed to detect the co-localization of ESCCAL-1 RNA and Gal-1 protein in ESCC cells. In brief, KYSE150 cells were seeded on the slides and fixed with 4% paraformaldehyde solution. After protease digestion and pre-hybridization, the cells were incubated overnight in hybridization solution containing CY3-labeled ESCCAL-1 probes. The cells were then subjected to BSA blocking, Gal-1 antibody (Abcam, USA) incubation, and fluorescence-labeled secondary antibody incubation. Finally, the cells were detected by fluorescence microscope following DAPI staining. The probe sequence is listed in Supplementary Table 1.

### Bioinformatics analysis

The expression profile data of LncRNAs obtained from the TCGA Project (https://xenabrowser.net/datapages) and GEO (https://www.ncbi.nlm.nih.gov/geo/) were used for differential gene expression analysis. Fold change (FC) >2.0 and *p* value < 0.01 was regarded as significant difference. The online tumor database GEPIA^52^ was used to analyze the transcription of ESCCAL-1 and Gal-1 in ESCA. The RNAfold Web Server was employed to predict secondary structure of ESCCAL-1 transcript. E3 ubiquitin ligases that potentially modify Gal-1 protein were predicted by UbiBrowser (http://ubibrowser.ncpsb.org.cn/ubibrowser/). The protein–protein interaction (PPI) network of Gal-1 was analyzed by STRING (https://www.string-db.org/).

### Statistical analysis

The data are presented as mean ± standard deviation (SD) and statistically analyzed by the SPSS 19.0 and Graphpad Prism 7.0. The student’s *t*-test was used to analyze the statistical differences between two groups. The expression correlation was determined by Pearson coefficient analysis. The diagnostic value of ESCCAL-1 in each cohort was presented as the receiver operating characteristic (ROC) curve. The correlation between ESCCAL-1 expression and clinicopathological features of ESCC patients was evaluated by Chi-square test. A *p* value < 0.05 was considered to be statistically significant.

### Reporting summary

Further information on research design is available in the Nature Research Reporting Summary linked to this article.

## Supplementary information

Supplementary Information

REPORTING SUMMARY

## Data Availability

The data that support the findings of this study are available from the corresponding author upon reasonable request. The public data of GSE120356, GSE53622, GSE53624, and GSE53625 are available from the GEO database (https://www.ncbi.nlm.nih.gov/geo/). The public data of TCGA-ESCA is available from the TCGA Project (https://xenabrowser.net/datapages).
