# Supplementary material for: ESCCAL-1 promotes cell-cycle progression by interacting with and stabilizing galectin-1 in esophageal squamous cell carcinoma
Source: NPJ Precis Oncol. 2022 Mar 1;6:12. doi: 10.1038/s41698-022-00255-x (PMC8888636; doi:10.1038/s41698-022-00255-x)

Supplementary Figures and Legends

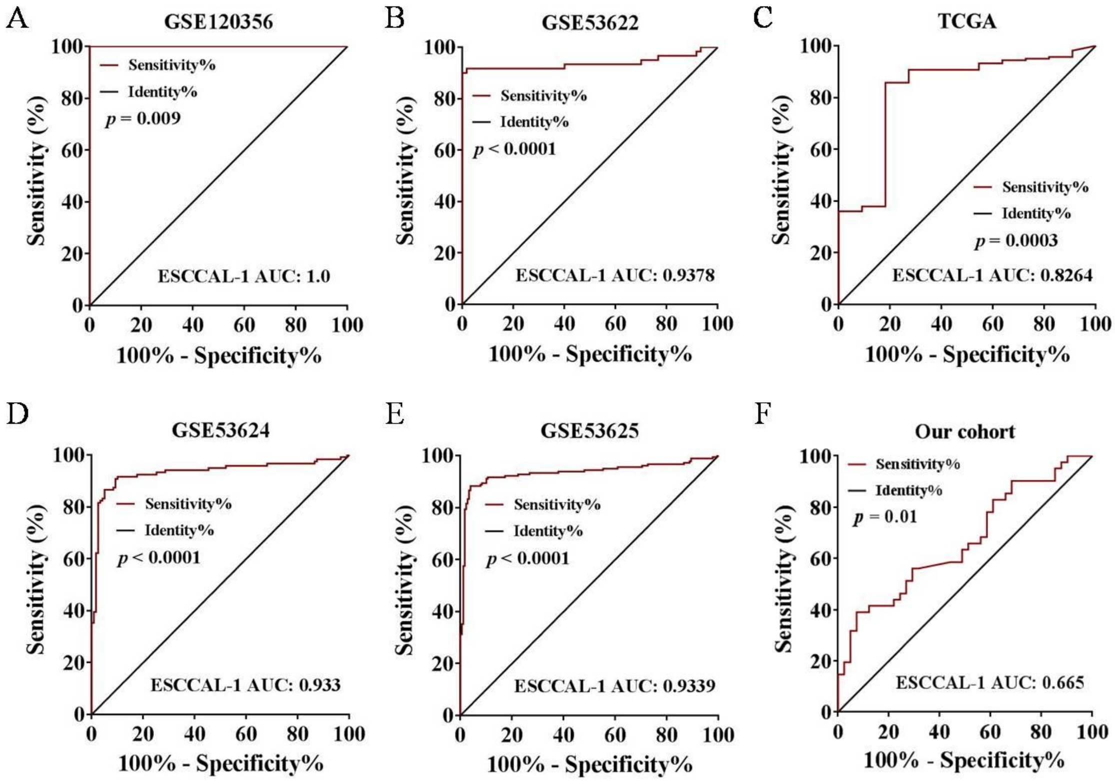

Supplementary Figure 1. The diagnostic values of ESCCAL-1 in multiple ESCA cohorts were evaluated by receiver operating characteristic (ROC) curves. (A-F) The larger area under the curve (AUC) indicated the higher diagnostic value.

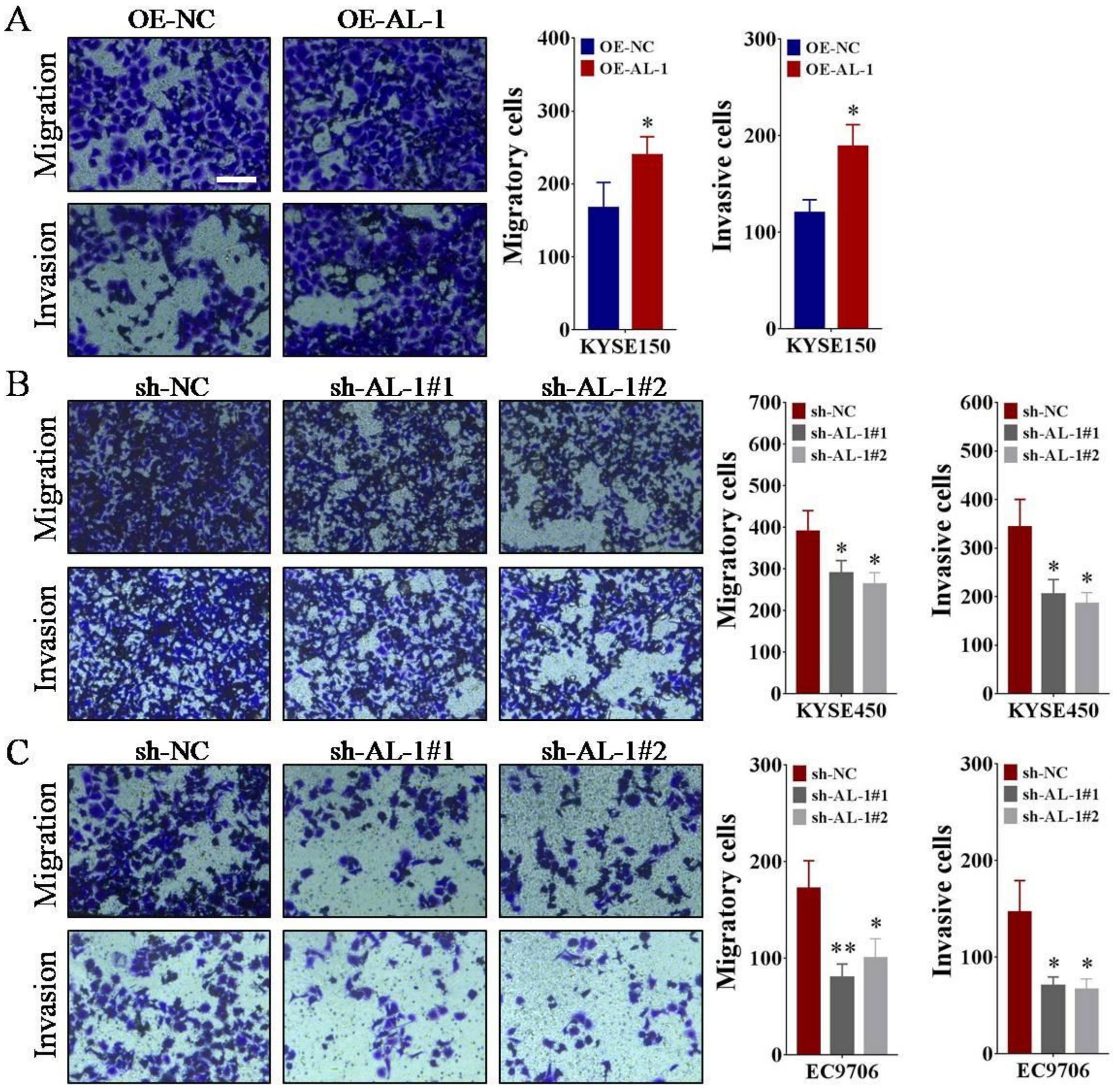

Supplementary Figure 2. Over-expression of ESCCAL-1 promotes ESCC migration and invasion *in vitro*. (A-C) Cell migration and invasion of ESCC after ESCCAL-1 over-expression (A) or knockdown (B-C) were tested with Transwell assays, *$p < 0.05$ as compared to OE-NC (A), *$p < 0.05$ or **$p < 0.01$ as compared to sh-NC (B-C). Scale bar = 100 μm.

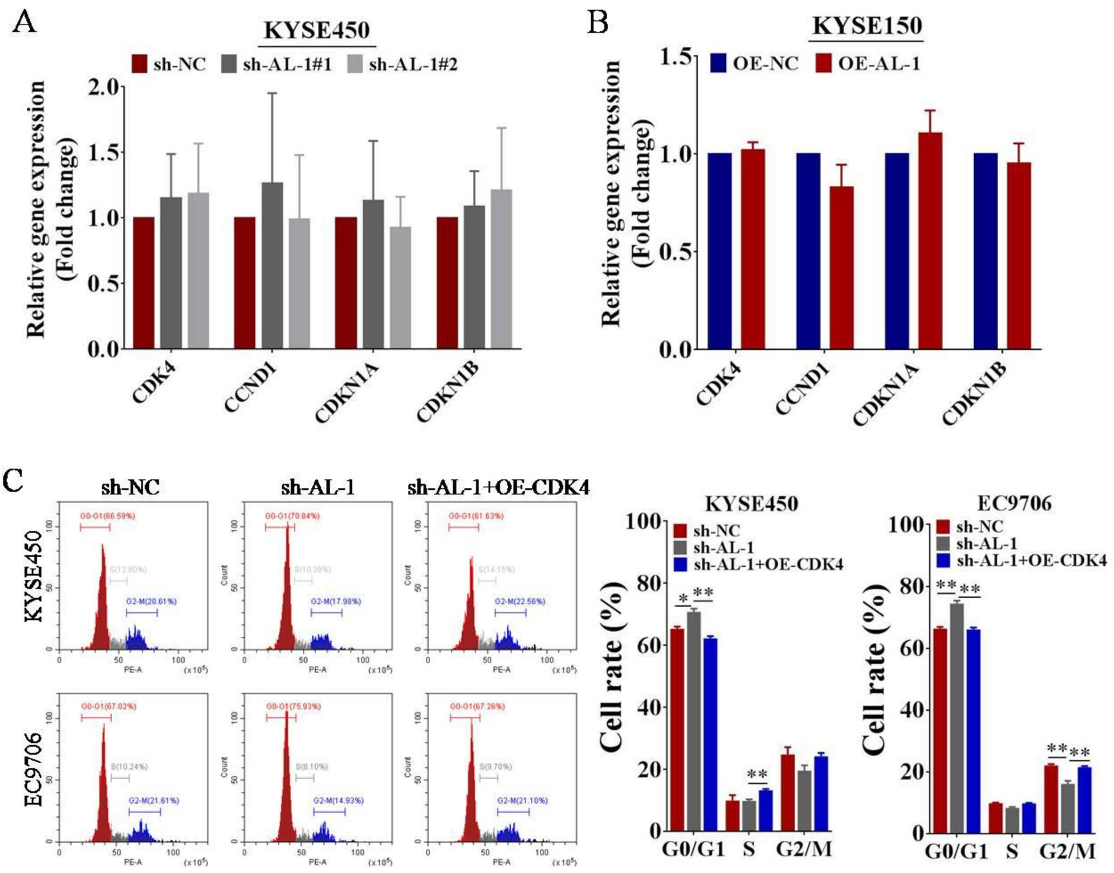

Supplementary Figure 3. The mRNA levels of cell-cycle regulators in ESCC cells following ESCCAL-1 manipulation. (A-B) The transcription of CDK4, CCND1, CDKN1A and CDKN1B in ESCC cells after ESCCAL-1 knockdown (A) or over-expression (B) were tested with qRT-PCR. (C) Flow cytometry was performed to examine cell-cycle changes after over-expression of CDK4 in ESCCAL-1-deficient ESCC cells, *$p < 0.05$ or **$p < 0.01$.

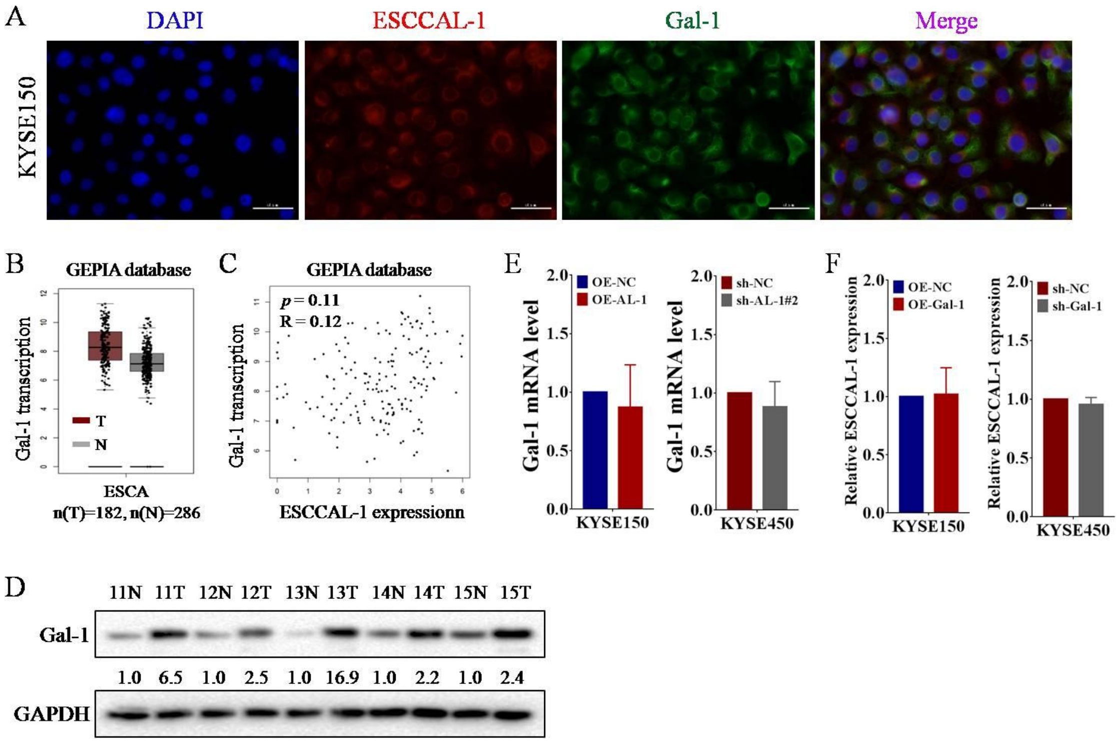

Supplementary Figure 4. The co-localization of ESCCAL-1 and Gal-1 in ESCC cells and the expression of Gal-1 in ESCC. (A) The immunofluorescence assay was utilized to detect the co-localization of ESCCAL-1 RNA and Gal-1 protein in KYSE150 cells. (B) The transcription of Gal-1 had no significant difference between 286 cases of normal tissues and 182 cases of ESCA tissues from the GEPIA dataset. (C) Pearson coefficient was used to analyze the correlation between ESCCAL-1 expression and Gal-1 mRNA level in 182 cases of ESCA tissues from the GEPIA dataset. (D) The protein levels of Gal-1 in 15 paired ESCC tumor tissues (T) and matched adjacent normal tissues (N) were assessed by Western blot (showing another 5 pairs). (E) The transcription of Gal-1 in ESCC cells after ESCCAL-1 over-expression or knockdown were tested with qRT-PCR. (F) The transcription of ESCCAL-1 in ESCC cells after Gal-1 over-expression or knockdown were tested with qRT-PCR.

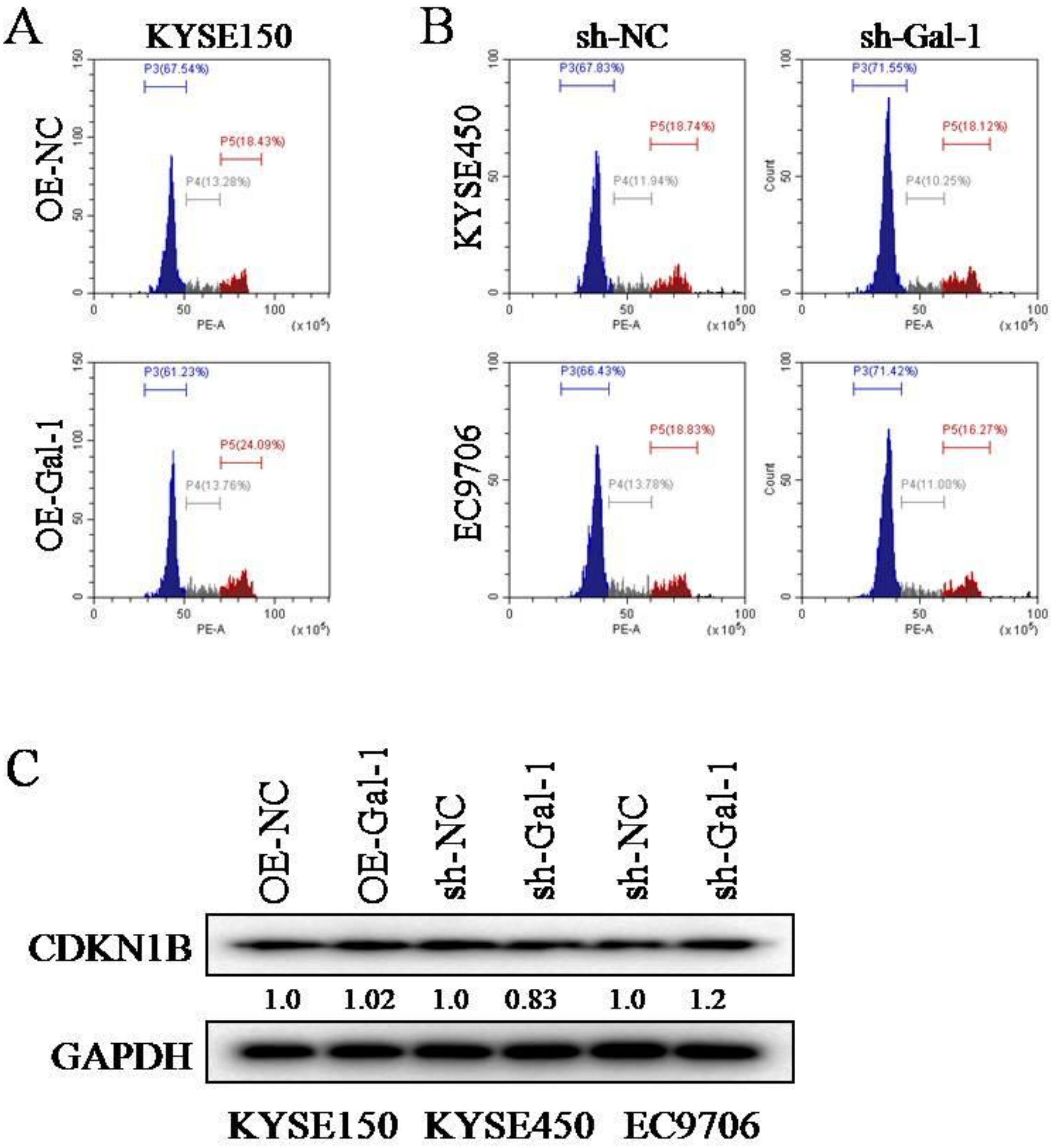

Supplementary Figure 5. Over-expression of Gal-1 promotes ESCC cell-cycle progression. (A-B) Flow cytometry was performed to examine the effects of Gal-1 manipulation on cell cycle of ESCC. Significant cell cycle promotion and inhibition were observed when Gal-1 was over-expressed (A) or knocked down (B). (C) The protein levels of CDKN1B in ESCC cells following Gal-1 over-expression or knockdown were determined by Western blot. Over-expression of Gal-1 has no significant effect on CDKN1B protein.

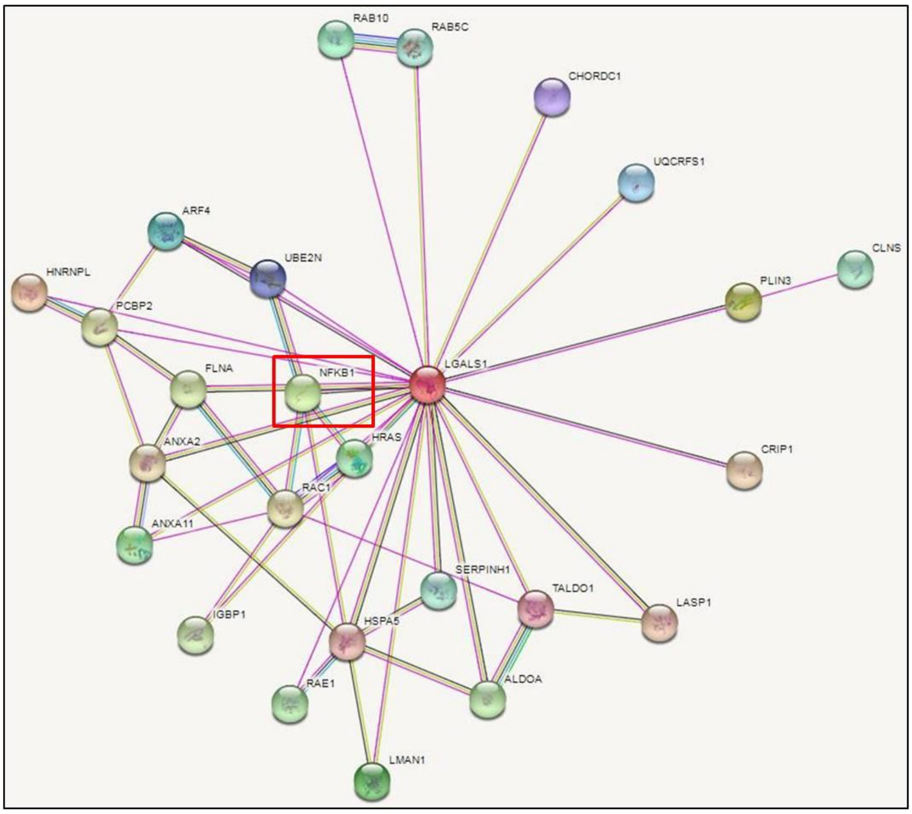

Protein-protein interaction (PPI) network of GAL1 analyzed by STRING

Supplementary Figure 6. NF-κB signaling may be a downstream pathway of Gal-1. The online tool STRING was used to analyze the potential downstream signaling pathways of Gal-1 through protein-protein interaction (PPI) network.

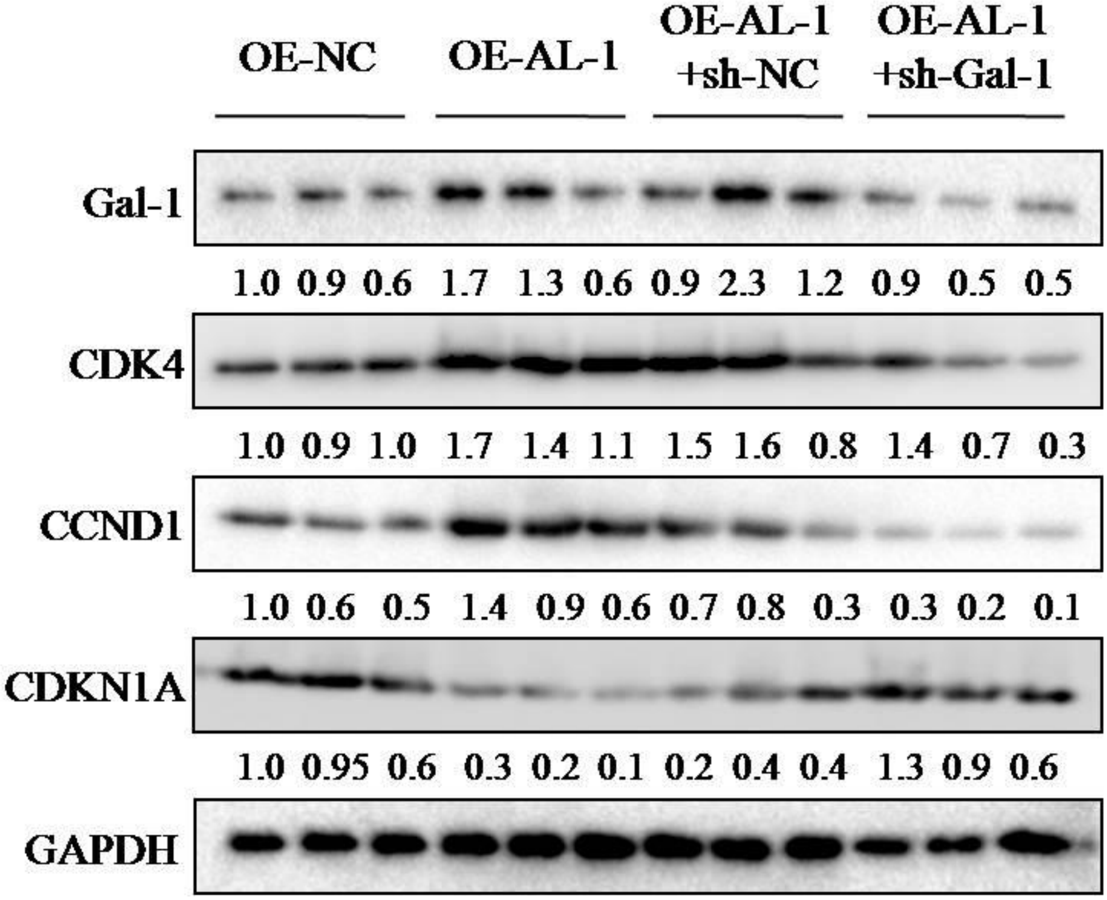

Supplementary Figure 7. The protein levels of cell-cycle regulators in tumors from nude mice. The protein levels of cell-cycle regulators (CDK4, CCND1 and CDKN1A) and Gal-1 in xenografts tumors were tested by Western blot.

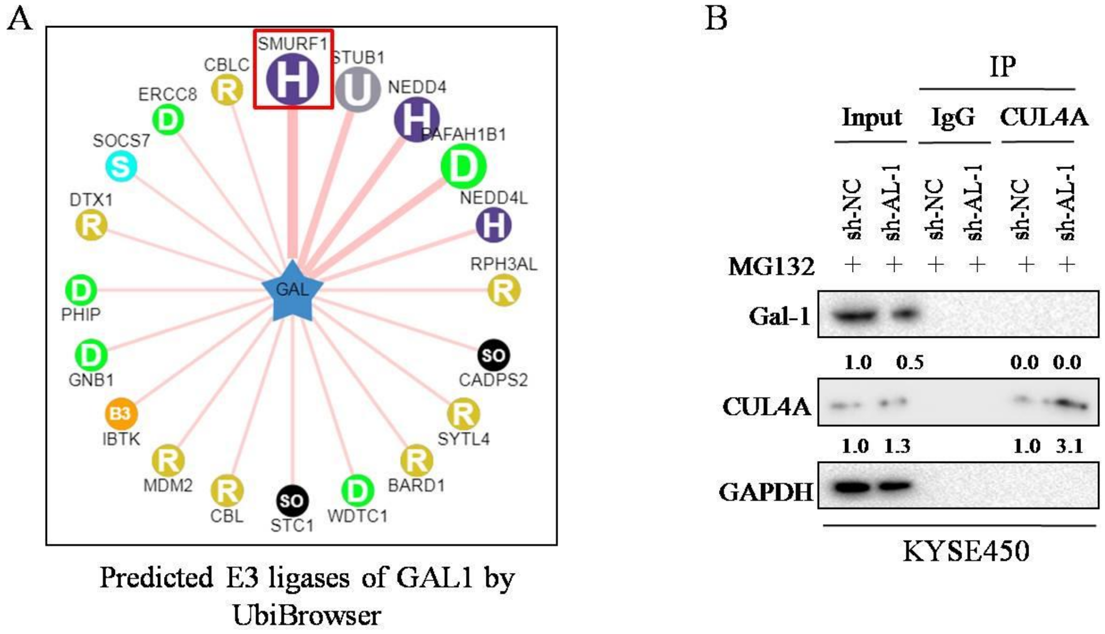

Supplementary Figure 8. Smurf1 but not CUL4A mediates the ubiquitination of Gal-1 in ESCC. (A) The online tool UbiBrowser was used to predict the potential E3 ligase that mediates Gal-1 ubiquitination modification. Smurf1 had the highest confidence among the predictions. (B) Co-IP combined with Western blot showed the unobserved interaction between CUL4A and Gal-1 protein in ESCC cells.

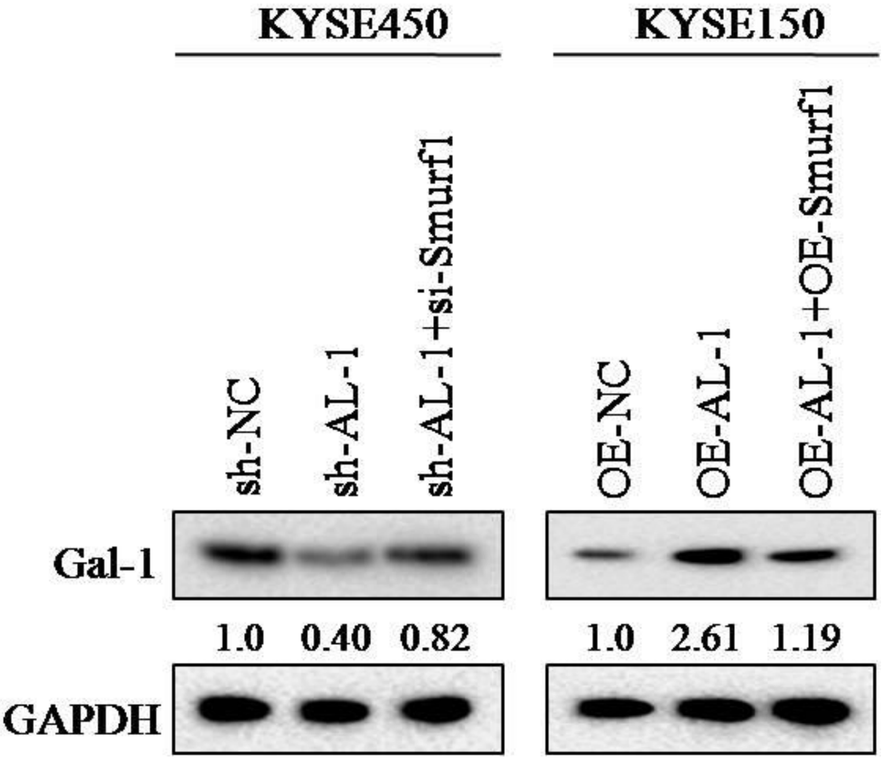

Supplementary Figure 9. The effects of ESCCAL-1/Smurf1 on Gal-1 levels. Knockdown or over-expression of Smurf1 removed the ESCCAL-1-dependent changes in Gal-1 protein level as examined by Western blot.

Supplementary Table 1

Primers for qRT-PCR

Name		Sequence (5' to 3')
ESCCAL-1	forward	CCAGACAGCAGCAAAGCAAT
	reverse	GGAAGCAGCAAATGTGTCCAT
Gal-1	forward	CTGTGCCTGCACTTCAACC
	reverse	CATCTGGCAGCTTGACGGT
GAPDH	forward	TCAGAGGACGGCATGAGACTTA
	reverse	AGCAGGACCCAGGTGTCATT
CDK4	forward	CGCCTATAATCCCAGCACGT
	reverse	CACCACCACGCCTGACTAAT
CCND1	forward	GGCGGAGGAGAACAAACAGA
	reverse	CTCCTCAGGTTCAGGCCTTG
CDKN1A	forward	TCTAGGAGGGAGACACTGGC
	reverse	TGTCTGACTCCTTGTTCCGC
CDKN1B	forward	GCCCTCCCCAGTCTCTCTTA
	reverse	CTCCCAAGCACCTCGGATTT

siRNAs sequence

Name		Sequence (5' to 3')
Negative control	sense	UUCUCCGAACGUGUCACGUTT
	antisense	ACGUGACACGUUCGGAGAATT
si-Smurf1#1	sense	CCAGUAUUCUACGGACAAUTT
	antisense	AUUGUCCGUAGAAUACUGGTT
si-Smurf1#2	sense	CAUGAAAUGCUGAAUCCUUTT
	antisense	AAGGAUUCAGCAUUUCAUGTT

probe sequence for ESCCAL-1

Name	Sequence (5' to 3')
ESCCAL-1 probe	CAGGUUGAAAAUGAUGUCGCUUGGCACAGUUCUC

Supplementary Table 2

Correlation between ESCCAL-1 expression and clinicopathological features of ESCC patients

Clinicopathological features	Total	ESCCAL-1 expression		Chi-square test
		Low	High	*P*-value
**Age (year)**				0.796
<60	11	5	6	
≥60	30	15	15	
**Gender**				0.228
Male	27	15	12	
Female	14	5	9	
**Differentiation**				0.007
Moderate	19	5	14	
High	22	15	7	
**Stage**				0.072
I-II	25	15	10	
III-IV	16	5	11	

Supplementary Table 3

Accession	Description
P61604	10 kDa heat shock protein, mitochondrial OS=Homo sapiens GN=HSPE1 PE=1 SV=2 - [CH10_HUMAN]
P09382	Galectin-1 OS=Homo sapiens GN=LGALS1 PE=1 SV=2 - [LEG1_HUMAN]
P06899	Histone H2B type 1-J OS=Homo sapiens GN=HIST1H2BJ PE=1 SV=3 - [H2B1J_HUMAN]
P62805	Histone H4 OS=Homo sapiens GN=HIST1H4A PE=1 SV=2 - [H4_HUMAN]
P10599	Thioredoxin OS=Homo sapiens GN=TXN PE=1 SV=3 - [THIO_HUMAN]
P07737	Profilin-1 OS=Homo sapiens GN=PFN1 PE=1 SV=2 - [PROF1_HUMAN]
P13645	Keratin, type I cytoskeletal 10 OS=Homo sapiens GN=KRT10 PE=1 SV=6 - [K1C10_HUMAN]
O14548	Cytochrome c oxidase subunit 7A-related protein, mitochondrial OS=Homo sapiens GN=COX7A2L PE=1 SV=2 - [C
P62750	60S ribosomal protein L23a OS=Homo sapiens GN=RPL23A PE=1 SV=1 - [RL23A_HUMAN]
P46783	40S ribosomal protein S10 OS=Homo sapiens GN=RPS10 PE=1 SV=1 - [RS10_HUMAN]
P35527	Keratin, type I cytoskeletal 9 OS=Homo sapiens GN=KRT9 PE=1 SV=3 - [K1C9_HUMAN]
P31949	Protein S100-A11 OS=Homo sapiens GN=S100A11 PE=1 SV=2 - [S10AB_HUMAN]
P02533	Keratin, type I cytoskeletal 14 OS=Homo sapiens GN=KRT14 PE=1 SV=4 - [K1C14_HUMAN]
O75964	ATP synthase subunit g, mitochondrial OS=Homo sapiens GN=ATP5L PE=1 SV=3 - [ATP5L_HUMAN]
P08779	Keratin, type I cytoskeletal 16 OS=Homo sapiens GN=KRT16 PE=1 SV=4 - [K1C16_HUMAN]
P35908	Keratin, type II cytoskeletal 2 epidermal OS=Homo sapiens GN=KRT2 PE=1 SV=2 - [K22E_HUMAN]
P58546	Myotrophin OS=Homo sapiens GN=MTPN PE=1 SV=2 - [MTPN_HUMAN]
P20674	Cytochrome c oxidase subunit 5A, mitochondrial OS=Homo sapiens GN=COX5A PE=1 SV=2 - [COX5A_HUMAN]
P39019	40S ribosomal protein S19 OS=Homo sapiens GN=RPS19 PE=1 SV=2 - [RS19_HUMAN]

Score	Coverage	# Proteins	# Unique Peptides	# Peptides	# PSMs	# AAs	MW [kDa]	calc. pI
739.38	57.84	1	7	7	30	102	10.9	8.92
369.37	32.59	1	5	5	17	135	14.7	5.50
276.89	31.75	14	2	5	11	126	13.9	10.32
213.82	31.07	1	3	3	11	103	11.4	11.36
241.64	29.52	1	3	3	8	105	11.7	4.92
243.58	28.57	1	3	3	8	140	15.0	8.27
2026.97	27.91	17	14	17	61	584	58.8	5.21
230.73	27.19	1	3	3	9	114	12.6	9.42
235.01	26.92	1	4	4	9	156	17.7	10.45
239.73	26.06	3	5	5	14	165	18.9	10.15
2071.99	25.36	2	15	16	71	623	62.0	5.24
422.32	23.81	1	3	3	15	105	11.7	7.12
1068.20	23.31	17	4	13	35	472	51.5	5.16
319.38	23.30	1	2	2	9	103	11.4	9.64
929.40	23.26	15	3	12	34	473	51.2	5.05
927.10	22.54	11	9	14	39	639	65.4	8.00
208.85	22.03	1	2	2	6	118	12.9	5.52
292.99	20.00	2	3	3	13	150	16.8	6.79
286.88	20.00	1	3	3	10	145	16.1	10.32

Supplementary materials: original images of blots in each Figure

Original blots of Fig. 3E, 3F, 3G

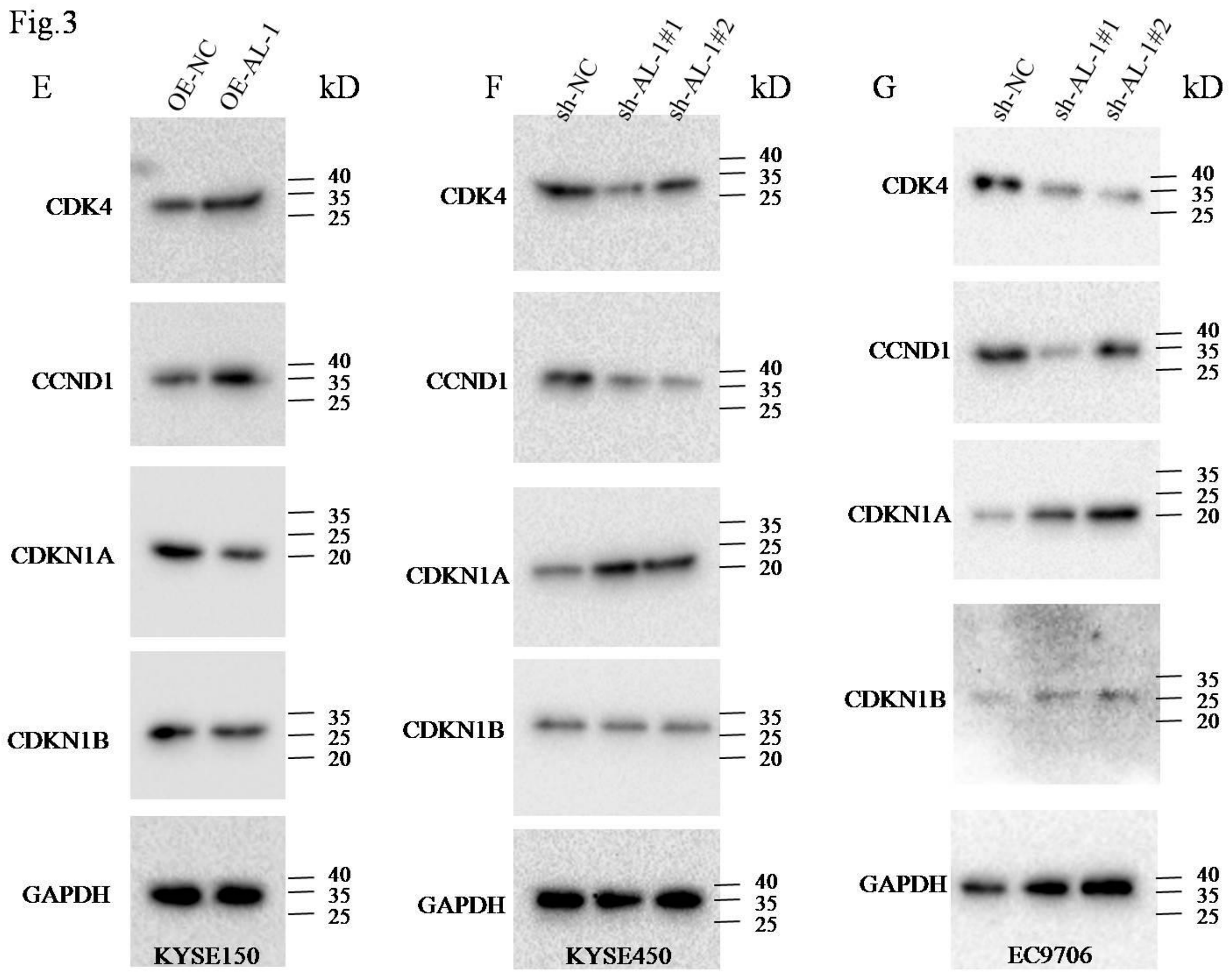

Original blots of Fig. 4C, 4F, 4H, 4J

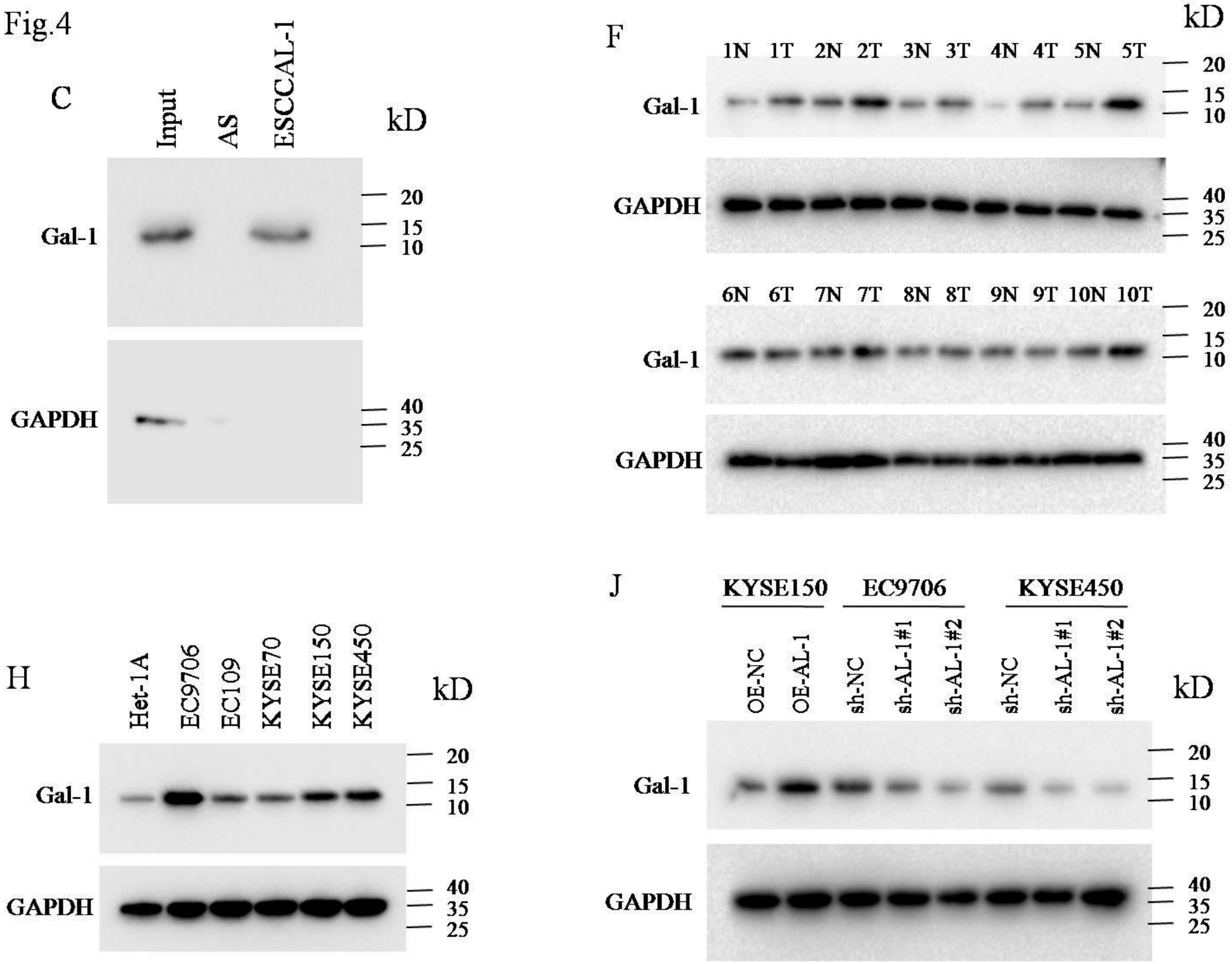

Original blots of Fig. 5A, 5K

Fig.5

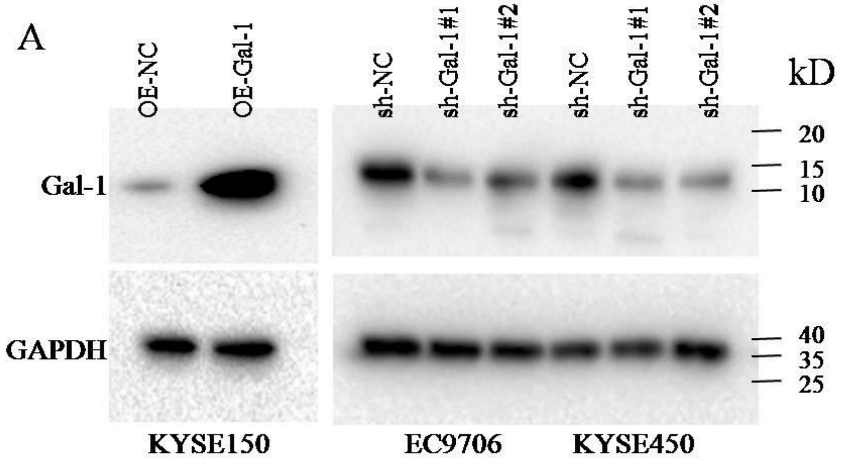

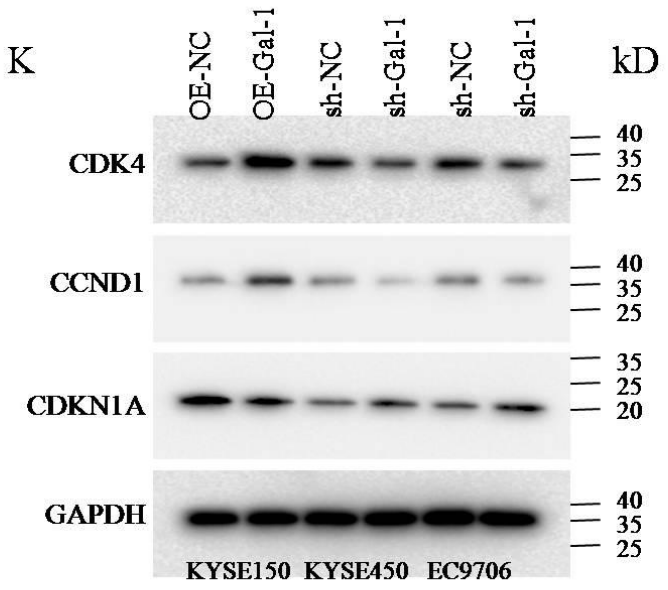

Original blots of Fig. 6D, 6E

Fig.6

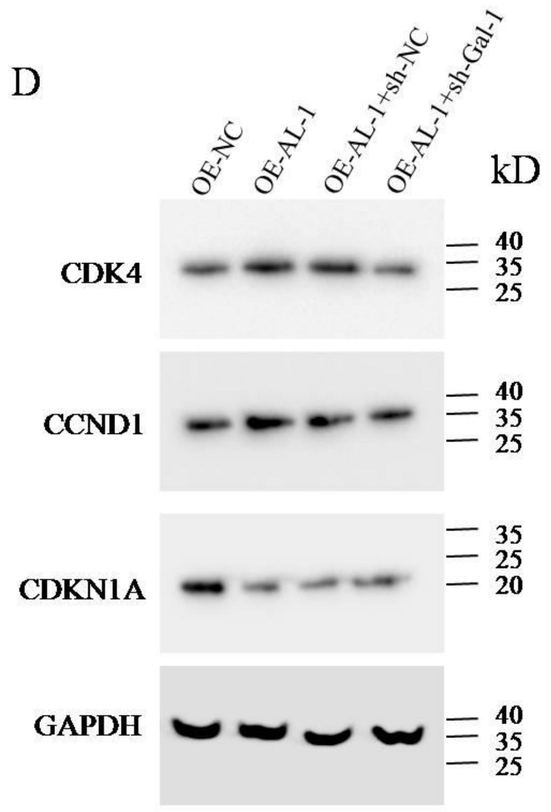

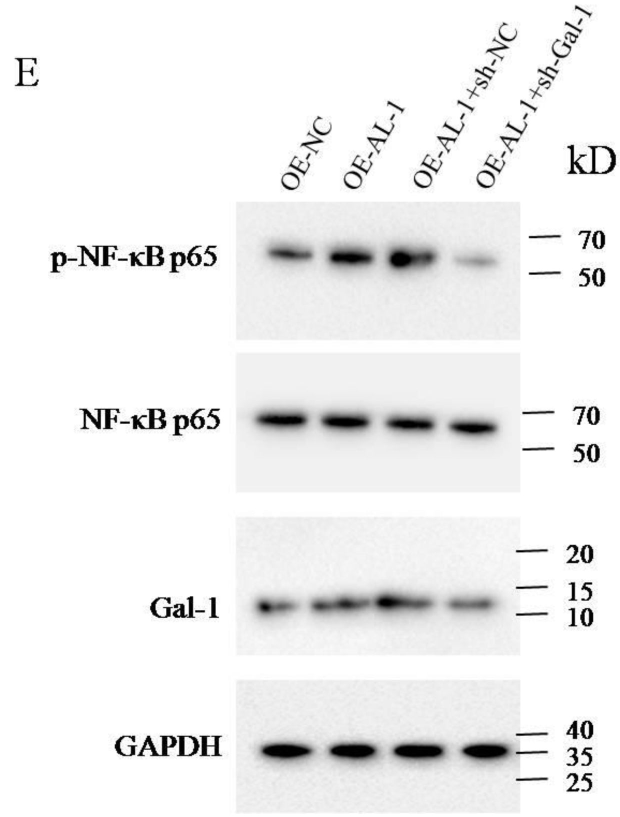

Original blots of Fig. 8A, 8B, 8C

Fig.8

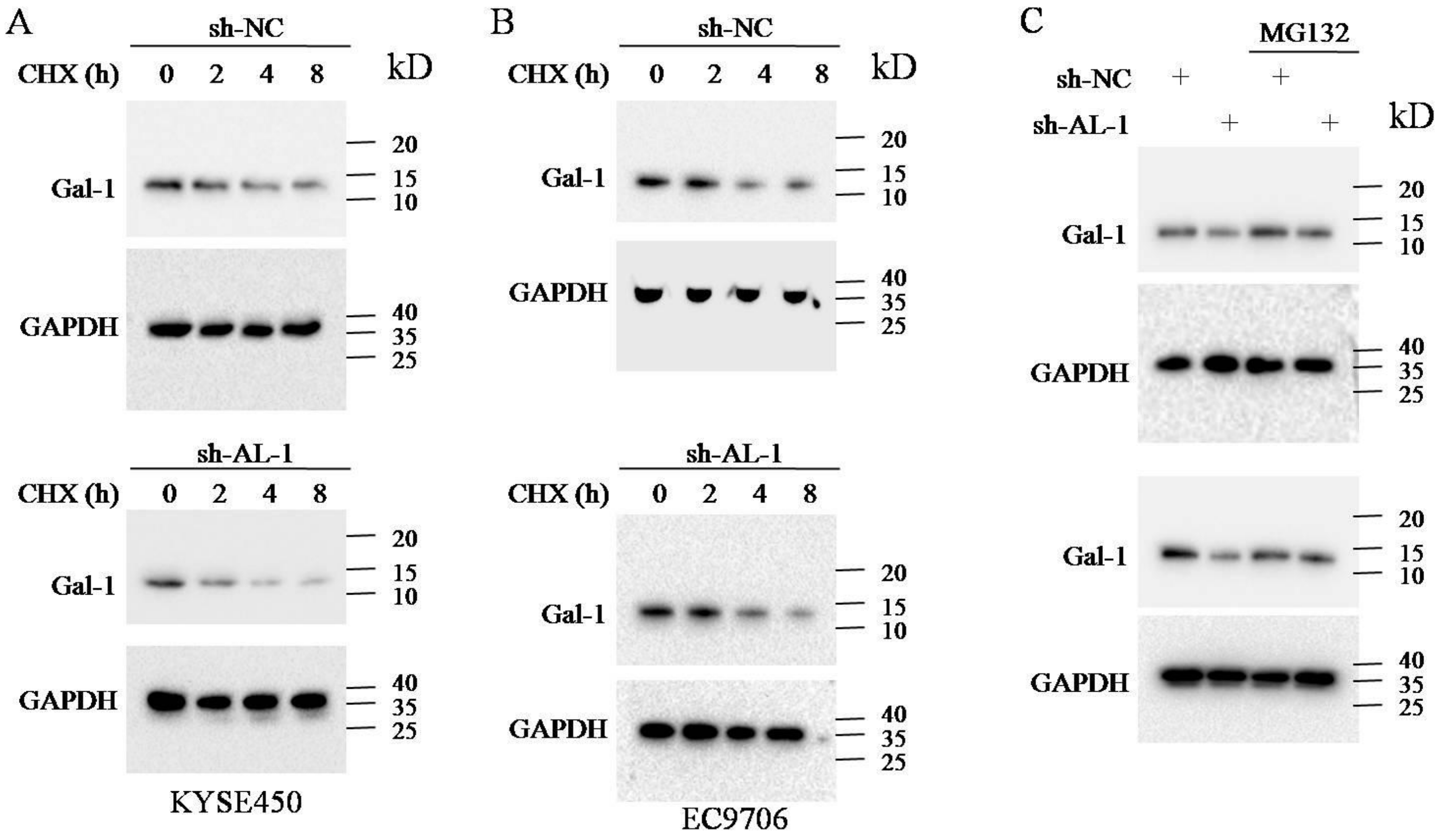

Original blots of Fig. 8D, 8E, 8F

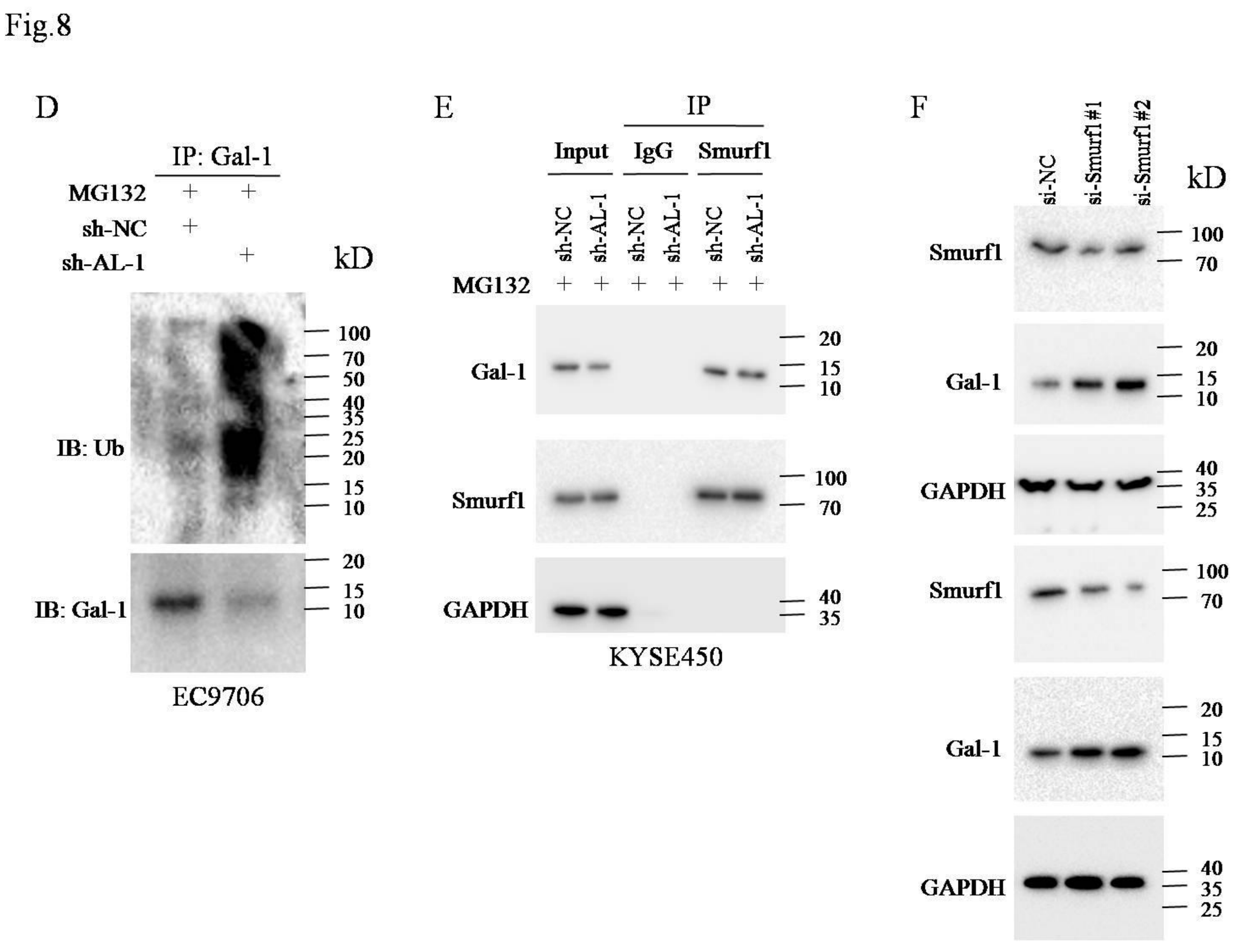

Original blots of Suppl. Fig. 4D

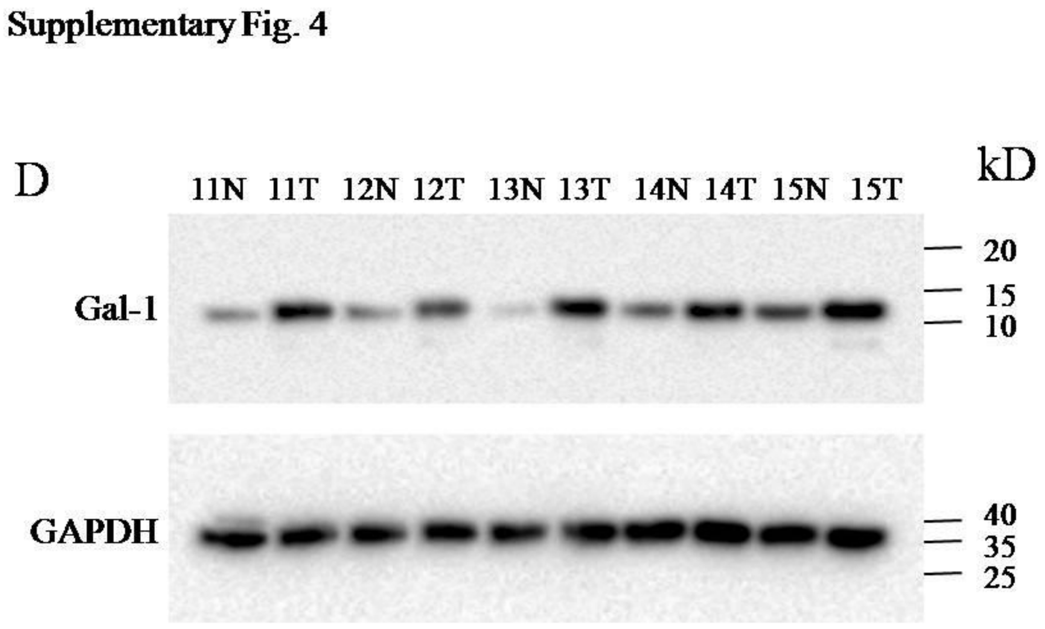

Original blots of Suppl. Fig. 5C

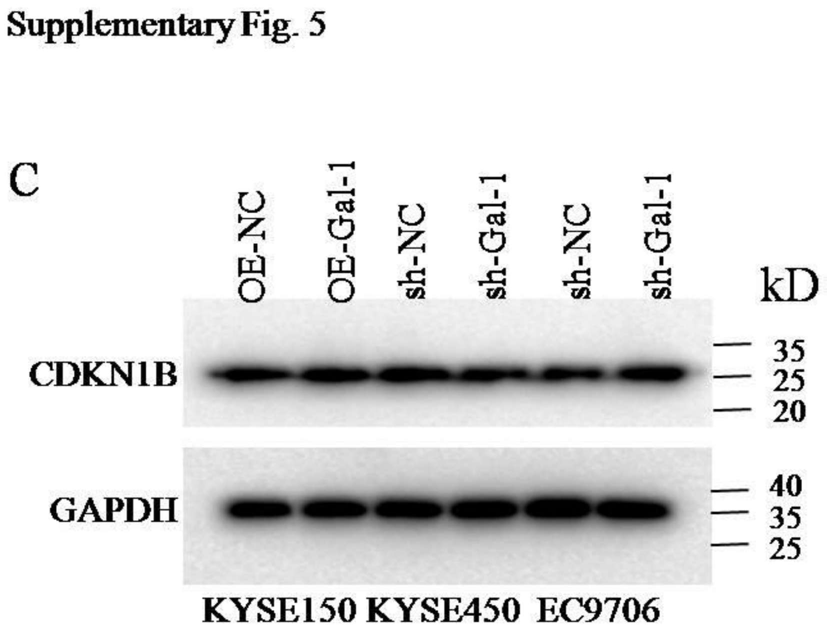

Original blots of Suppl. Fig. 7

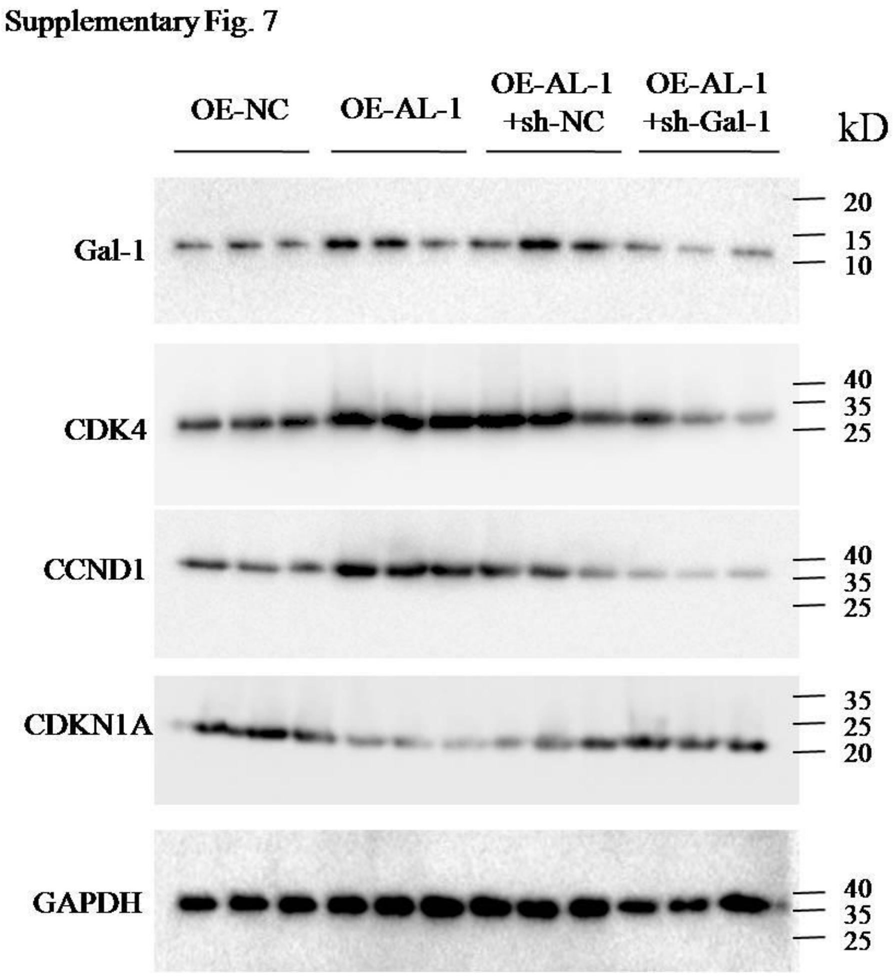

Original blots of Suppl. Fig. 8B

Supplementary Fig. 8

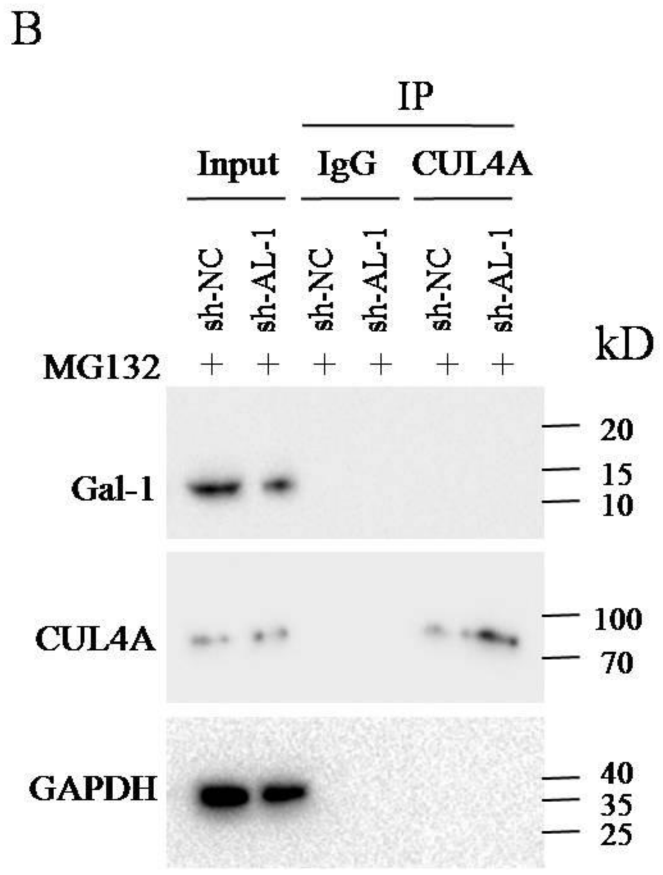

Original blots of Suppl. Fig. 9

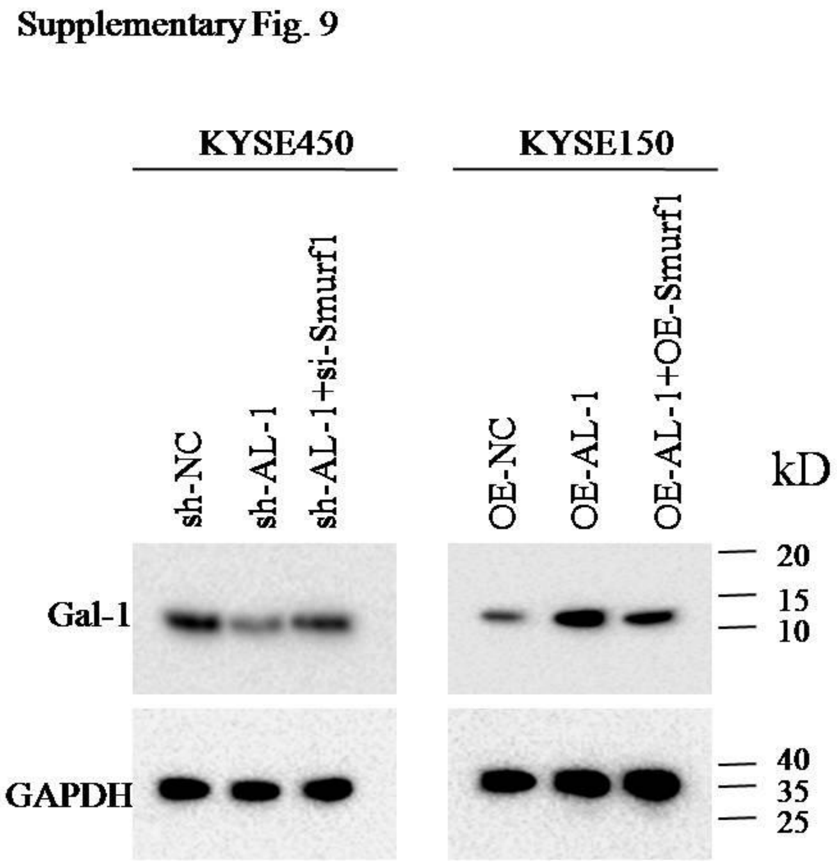

Supplement: Supplementary file 1 — Supplementary Information [file 41698_2022_255_MOESM1_ESM.pdf]
