# Supplementary material for: ESCCAL-1 promotes cell-cycle progression by interacting with and stabilizing galectin-1 in esophageal squamous cell carcinoma
Source: NPJ Precis Oncol. 2022 Mar 1;6:12. doi: 10.1038/s41698-022-00255-x (PMC8888636; doi:10.1038/s41698-022-00255-x)
Supplement: Supplementary file 2 — REPORTING SUMMARY [file 41698_2022_255_MOESM2_ESM.pdf]

nature portfolio

Corresponding author(s): DBPR,NPJPRECISIONONCOLOGY-01431R

Last updated by author(s): Jan 14, 2022

Reporting Summary

Nature Portfolio wishes to improve the reproducibility of the work that we publish. This form provides structure for consistency and transparency in reporting. For further information on Nature Portfolio policies, see our Editorial Policies and the Editorial Policy Checklist.

Statistics

For all statistical analyses, confirm that the following items are present in the figure legend, table legend, main text, or Methods section.

n/a	Confirmed	
☐	☒	The exact sample size (*n*) for each experimental group/condition, given as a discrete number and unit of measurement
☐	☒	A statement on whether measurements were taken from distinct samples or whether the same sample was measured repeatedly
☐	☒	The statistical test(s) used AND whether they are one- or two-sided<br>*Only common tests should be described solely by name; describe more complex techniques in the Methods section.*
☐	☒	A description of all covariates tested
☐	☒	A description of any assumptions or corrections, such as tests of normality and adjustment for multiple comparisons
☐	☒	A full description of the statistical parameters including central tendency (e.g. means) or other basic estimates (e.g. regression coefficient) AND variation (e.g. standard deviation) or associated estimates of uncertainty (e.g. confidence intervals)
☐	☒	For null hypothesis testing, the test statistic (e.g. *F*, *t*, *r*) with confidence intervals, effect sizes, degrees of freedom and *P* value noted<br>*Give P values as exact values whenever suitable.*
☒	☐	For Bayesian analysis, information on the choice of priors and Markov chain Monte Carlo settings
☒	☐	For hierarchical and complex designs, identification of the appropriate level for tests and full reporting of outcomes
☐	☒	Estimates of effect sizes (e.g. Cohen's *d*, Pearson's *r*), indicating how they were calculated

Our web collection on statistics for biologists contains articles on many of the points above.

Software and code

Policy information about availability of computer code

Data collection	The public data of GSE120356, GSE53622, GSE53624, and GSE53625 are obtained from the GEO database (https://www.ncbi.nlm.nih.gov/geo/). The public data of TCGA-ESCA is obtained from the TCGA Project (https://xenabrowser.net/datapages).
Data analysis	The expression profile data of LncRNAs obtained from the TCGA Project (https://xenabrowser.net/datapages) and GEO (https://www.ncbi.nlm.nih.gov/geo/) were used for differential gene expression analysis. Fold change (FC) greater than 2.0 and p value less than 0.01 was regarded as significant difference. The online tumor database GEPIA was used to analyze the transcription of ESCCAL-1 and Gal-1 in ESCA.

For manuscripts utilizing custom algorithms or software that are central to the research but not yet described in published literature, software must be made available to editors and reviewers. We strongly encourage code deposition in a community repository (e.g. GitHub). See the Nature Portfolio guidelines for submitting code & software for further information.

Data

Policy information about availability of data

All manuscripts must include a data availability statement. This statement should provide the following information, where applicable:

- Accession codes, unique identifiers, or web links for publicly available datasets
- A description of any restrictions on data availability
- For clinical datasets or third party data, please ensure that the statement adheres to our policy

The data that support the findings of this study are available from the corresponding author upon reasonable request.

Field-specific reporting

Please select the one below that is the best fit for your research. If you are not sure, read the appropriate sections before making your selection.

☒ Life sciences ☐ Behavioural & social sciences ☐ Ecological, evolutionary & environmental sciences

For a reference copy of the document with all sections, see nature.com/documents/nr-reporting-summary-flat.pdf

Life sciences study design

All studies must disclose on these points even when the disclosure is negative.

Sample size	In this study, no sample size calculation was performed. In order to clarify the dysregulated status of ESCCAL-1 in ESCC tumors, we included 7 additional independent ESCC study cohorts for validation, including 41 newly collected ESCC tissue specimens (our cohort) and 6 public ESCC study cohorts, including TCGA, GEPIA, GSE120356, GSE53622, GSE53624, and GSE53625 datasets. All these results consistently show that ESCCAL-1 is substantially up-regulated in ESCC tumors as compared to normal tissues, providing strong evidence for clinical targeting of ESCCAL-1. We believe that these results are sufficient to demonstrate the dysregulation of ESCCAL-1 expression in ESCC.
Data exclusions	No data were excluded from the analyses.
Replication	The experiments were repeated two to four times to verify the reproducibility.
Randomization	The experimental samples in this study did not involve random grouping.
Blinding	Blinding was not relevant to this study, because in most cases, the experimental operation and data analysis are performed by the same investigator.

Reporting for specific materials, systems and methods

We require information from authors about some types of materials, experimental systems and methods used in many studies. Here, indicate whether each material, system or method listed is relevant to your study. If you are not sure if a list item applies to your research, read the appropriate section before selecting a response.

Materials & experimental systems

n/a	Involved in the study	
☐	☒	Antibodies
☐	☒	Eukaryotic cell lines
☒	☐	Palaeontology and archaeology
☐	☒	Animals and other organisms
☒	☐	Human research participants
☒	☐	Clinical data
☒	☐	Dual use research of concern

Methods

n/a	Involved in the study	
☒	☐	ChIP-seq
☐	☒	Flow cytometry
☒	☐	MRI-based neuroimaging

Antibodies

Antibodies used	Antibodies include anti-GAPDH (Bioworld, China, AP0063), anti-CCND1 (Bioworld, China, BS6532), anti-CDK4 (Bioworld, China, BS6462), anti-CDKN1A (Bioworld, China, BS6561), anti-CDKN1B (Bioworld, China, BS91000), anti-NF-κB p65 (Abcam, USA, ab32536), anti-p-NF-κB p65 (Abcam, USA, ab194726), anti-Galectin-1 (Santa Cruz, USA, sc-166618), anti-Ubiquitin (Santa Cruz, USA, sc-8017), anti-Smurf1 (Santa Cruz, USA, SC-100616), anti-CUL4A (Abnova, China, PAB20038).
Validation	The application of antibody was carried out according to the instructions, including applicable species, dilution ratio, etc.

Eukaryotic cell lines

Policy information about cell lines

Cell line source(s)	Two ESCC cell lines EC9706 and EC109 were previously obtained from the Cell Bank of Shanghai Academy of Biological Sciences. One normal esophagus epithelial cell line Het-1A and three ESCC cell lines KYSE70, KYSE150 and KYSE450 were kindly provided by the Bioengineering and Transformation Laboratory of Zhengzhou University.
Authentication	The identification of ESCC cell lines was carried out by short tandem repeat (STR) detection.
Mycoplasma contamination	All cell lines tested negative for mycoplasma contamination.

Commonly misidentified lines (See ICLAC register)	None.

Animals and other organisms

Policy information about studies involving animals; ARRIVE guidelines recommended for reporting animal research

Laboratory animals	6-week-old male BALB/c nude mice were used for in vivo experiments.
Wild animals	This study did not involve wild animals.
Field-collected samples	This study did not involve samples collected from the field.
Ethics oversight	The study was approved by the Ethics Committee of the Zhengzhou Central Hospital Affiliated to Zhengzhou University.

Note that full information on the approval of the study protocol must also be provided in the manuscript.

Flow Cytometry

Plots

Confirm that:

☒ The axis labels state the marker and fluorochrome used (e.g. CD4-FITC).

☒ The axis scales are clearly visible. Include numbers along axes only for bottom left plot of group (a 'group' is an analysis of identical markers).

☒ All plots are contour plots with outliers or pseudocolor plots.

☒ A numerical value for number of cells or percentage (with statistics) is provided.

Methodology

Sample preparation	ESCC cells were fixed with 70% ethanol at 4°C for 2 h, and subsequently reacted in staining buffer containing PI (7sea biotech, China) and RNase at 37°C for 30 min. Cell cycle was finally tested by a Flow Cytometer (Bechman, USA).
Instrument	Flow Cytometer (Bechman, USA, CytoFLEX System B2-R2-VO).
Software	CytExpert v2.3.1
Cell population abundance	ESCC cell lines were used. There is not cell sorting in this experiment
Gating strategy	FACS gating strategy for cell-cycle detection, first plot gating for living cells, then second plot for single cells. Double DNA content was g0-G1 phase, haploid DNA content was G2-M phase, and between the two was S phase.

☐ Tick this box to confirm that a figure exemplifying the gating strategy is provided in the Supplementary Information.
